# Hepatic lipid signatures of little brown bats (*Myotis lucifugus*) and big brown bats (*Eptesicus fuscus*) at early stages of white-nose syndrome

**DOI:** 10.1038/s41598-021-90828-w

**Published:** 2021-06-02

**Authors:** Evan L. Pannkuk, Nicole A. S.-Y. Dorville, Yvonne A. Dzal, Quinn E. Fletcher, Kaleigh J. O. Norquay, Craig K. R. Willis, Albert J. Fornace, Evagelia C. Laiakis

**Affiliations:** 1 Department of Oncology, Lombardi Comprehensive Cancer Center, Georgetown University Medical Center, Washington, DC 20057 USA; 2 Department of Biology and Centre for Forest Interdisciplinary Research (C-FIR), University of Winnipeg, Winnipeg, MB Canada; 3 Department of Biochemistry and Molecular and Cellular Biology, Georgetown University Medical Center, Washington, DC 20057 USA

**Keywords:** Biochemistry, Lipidomics, Metabolomics

## Abstract

White-nose syndrome (WNS) is an emergent wildlife fungal disease of cave-dwelling, hibernating bats that has led to unprecedented mortalities throughout North America. A primary factor in WNS-associated bat mortality includes increased arousals from torpor and premature fat depletion during winter months. Details of species and sex-specific changes in lipid metabolism during WNS are poorly understood and may play an important role in the pathophysiology of the disease. Given the likely role of fat metabolism in WNS and the fact that the liver plays a crucial role in fatty acid distribution and lipid storage, we assessed hepatic lipid signatures of little brown bats (*Myotis lucifugus*) and big brown bats (*Eptesicus fuscus*) at an early stage of infection with the etiological agent, *Pseudogymnoascus destructans* (Pd). Differences in lipid profiles were detected at the species and sex level in the sham-inoculated treatment, most strikingly in higher hepatic triacylglyceride (TG) levels in *E. fuscus* females compared to males. Interestingly, several dominant TGs (storage lipids) decreased dramatically after Pd infection in both female *M. lucifugus* and *E. fuscus*. Increases in hepatic glycerophospholipid (structural lipid) levels were only observed in *M. lucifugus*, including two phosphatidylcholines (PC [32:1], PC [42:6]) and one phosphatidylglycerol (PG [34:1]). These results suggest that even at early stages of WNS, changes in hepatic lipid mobilization may occur and be species and sex specific. As pre-hibernation lipid reserves may aid in bat persistence and survival during WNS, these early perturbations to lipid metabolism could have important implications for management responses that aid in pre-hibernation fat storage.

## Introduction

White-nose syndrome (WNS) is a cutaneous fungal infection responsible for unprecedented mortality of many North American hibernating bat species^1^. The etiological agent, *Pseudogymnoascus destructans* (Pd) colonizes bat integument during hibernation, invades sweat and sebaceous glands, and causes visible epidermal necrosis and lesions to wing tissues^2,3^. Wing membrane damage leads to downstream physiological complications, such as increased electrolyte depletion^4^ and inflammation^5^. In addition, bats exhibit more frequent torpor arousals^6,7^ during WNS and increased metabolic rates are observed at several disease stages including early infection, torpor, and subsequent healing^8–10^. Increased metabolism and increased torpor arousals contribute to premature fat depletion during winter months, when food availability is low, and may be a primary driver of Pd-related mortality^11^. Interspecific susceptibility to WNS varies, as little brown bats (*Myotis lucifugus*) appear to be much more sensitive and in danger of long-term population declines compared to big brown bats (*Eptesicus fuscus*), likely due to several life history factors^11^. Shifts in relative abundance of these two species indicate drastic decreases for *M. lucifugus* (− 79.6% [Midwestern site, IN], − 99% [S. Appalachian site, NC/TN]) while *E. fuscus* has remained relatively stable (+ 11.5% [Midwestern site, IN], + 10% [S. Appalachian site, NC/TN])^12,13^. Also, intraspecific susceptibility may vary as female bats conserve fat reserves through winter months compared to males to increase fecundity in spring^14^. Although past studies suggest that lipid metabolism and premature fat depletion play an important role in Pd-related mortality, details on lipid mobilization during the early and late phases of WNS are lacking. Specifically, inter- and intraspecific differences in lipid metabolism during WNS are poorly understood and may play an important role in WNS pathophysiology and bat overwinter survival.

Lipids are broadly defined as small molecules that are hydrophobic or amphipathic in nature that can be classified into eight categories, including (1) fatty acyls, (2) glycerolipids (GLs, e.g., diacylglyceride), (3) glycerophospholipids (GPs), (4) sphingolipids, (5) sterols, (6) prenols, (7) saccharolipids, and (8) polyketides, with further subdivisions based on type of head group and aliphatic chain linkages^15^. Functionally, lipids play several roles that include maintaining cellular structure (e.g., GPs, sphingomyelins, glycolipids), signaling (e.g., oxidized lipids, ceramides, LysoGPs), and storage/energy (e.g., fatty acids and GLs)^16^. For energy storage, lipids are assembled into lipid droplets that are composed of a neutral lipid (triacylglycerides [TGs] and cholesteryl esters [CEs]) core surrounded by a structural GP monolayer and associated proteins^17^. Hibernators utilize these stored TG reserves for energy during winter months, where they are hydrolyzed to free fatty acids and subsequently metabolized in the liver through fatty acid β-oxidation or repackaged and transported to other tissues as very low-density lipoproteins (VLDL)^18^. Lipid mobilization from tissues primarily involved in lipid metabolism (liver, white adipose tissue, brown adipose tissue) through enzymatic hydrolysis affects the chemical composition, size, and distribution of these lipid droplets and has been shown to influence migration and exercise performance^19–21^. Intramuscular fat stores may also play important roles in subsequent shivering thermogenesis^22^. Therefore, determining their lipid composition and defining fat quality during WNS may aid in deciphering differential metabolic consequences of Pd infection and the utility of pre-hibernation fat strategies.

Recent developments in analytical techniques have allowed researchers to obtain relative quantification and structural data on hundreds of lipid compounds (termed lipidomics) for quickly assessing changes in tissue lipid composition^23^. Lipidomic analyses within bats (order Chiroptera) has remained rather specialized, and has largely focused on integumentary lipids and their role in cutaneous water loss^24,25^, intraspecific communication^26^, and lipid composition after Pd infection^27,28^, along with more targeted approaches assessing species specificity and resistance to Pd^29–31^. Additional lipidomic studies beyond bat integument have focused on individual animal lifespan^32,33^. However, less attention has been placed on changes in lipid metabolism beyond percentage of total body fat levels post Pd infection^11,34,35^.

In this study, we assessed changes in lipid signatures in *M. lucifugus* (WNS-sensitive) and *E. fuscus* (less susceptible to WNS) at an early stage of Pd infection. Given the importance of the liver in lipid metabolism and fatty acid “shuttling”, we focused on changes in hepatic lipid levels. As metabolic perturbations and fat depletion play a major role in Pd-related mortality, our hypothesis is that inter- and intraspecific lipid metabolism may relate to overall body condition and contribute to survival. We predicted that after Pd infection: (1) *M. lucifugus* would show greater perturbation to overall hepatic lipid levels (relative to sham-treatment) compared to *E. fuscus* and (2) as female bats conserve fat reserves through winter compared to males, male bats would have lower GL levels compared to females. To address these predictions *M. lucifugus* and *E. fuscus* (male and female) were inoculated with Pd or sham-inoculated, liver tissue was collected early in disease progression, and global hepatic lipid profiles were obtained using Ultra Performance Liquid Chromatography (UPLC) quadrupole time-of-flight (QTOF) mass spectrometry (MS). This is the first study to assess possible dyslipidemia and perturbations in lipid metabolism in liver tissue of bats at the early stages of Pd-infection.

## Materials and methods

### Animal model

Laboratory experiments were conducted at the University of Winnipeg (we confirm that all methods and experimental protocols were carried out in accordance with relevant guidelines and regulations, approved by institutional and/or licensing committees, and are in compliance with ARRIVE guidelines: Manitoba Sustainable Development Species at Risk/Wildlife Scientific Permit # SAR16009, the Ontario Ministry of Natural Resources Wildlife Scientific Collector’s Authorization 1,085,301 and University of Winnipeg Animal Care Protocol #AE08399). *M. lucifugus* were collected from a WNS-negative hibernaculum in Central Manitoba on 8 January (mid-winter) 2017. We swabbed 22 M*. lucifugus* (12 male, 10 female) individuals for Pd and swabbed the hibernaculum substrate prior to experimental Pd exposure. None of these samples tested positive for WNS. We collected 16 *E. fuscus* (8 male, 8 female) individuals from a hibernaculum in northwestern Ontario near Kenora on 18 January 2017 (described in^36^). We chose this *E. fuscus* hibernaculum because it was > 350 km from the nearest known WNS-positive site at the time^37^ and our surveillance indicated that it was negative for Pd the year prior to our study. Unfortunately, after we sampled *E. fuscus* from this site, Pd was detected there for the first time during winter 2017, but there was no visual evidence of WNS in the hibernaculum, and no *E. fuscus* had clinical signs of WNS at the time of capture. After the beginning of the experiment we found 3 *E. fuscus* used in this study tested positive for extremely low levels of Pd prior to treatment (Ct values from real-time and standard TaqMan assay quantitative polymerase chain reaction (qPCR) (Ct values: 39.41 ± 0.08)^38,39^. However, the contaminated bats likely had a negligible influence on the study as total load was near limit of detection for qPCR and substantially lower than our Pd treatment group at the end of the experiment (~ 18-fold lower, File S1).

Hibernating bats were removed from the cave wall by hand and transferred to a bio-secure bat facility at the University of Winnipeg, as previously described^36^. Bats were kept at 8 °C and 98% humidity in mesh cages (Exo-terra Flexarium; 22 cm × 35 cm × 43 cm) within temperature/humidity-controlled incubators (Caron Environmental Chamber model 6041; 90.1 cm × 84.5 cm × 228.9 cm) to encourage hibernation. *Eptesicus fuscus* were housed in two cages (one sham treatment, one Pd-inoculated: 8 individuals/cage, 4 male and 4 female) (File S2). *Myotis lucifugus* were housed in two cages (one sham treatment: 12 individuals/cage, 6 male and 6 female; one Pd-inoculated; 10 individuals/cage, 6 male and 4 female). Bats were infected with a Pd inoculum (20 μl, phosphate buffered saline with *Tween20* containing 5 × 10^5^ conidia) or sham inoculum (20 μl, phosphate buffered saline with *Tween20*) onto the wing and tail membranes. Animals were monitored with motion-activated infrared video (Digital Watchdog VMAX series) on a weatherproof, dome camera (Speco Technologies, model HD5941T) to quantify arousal frequency and monitor sickness behavior. Bats found unresponsive or extremely sluggish were humanely euthanized (CO_2_ inhalation after isoflurane anesthesia). At the same time as the current study, we were also running an experiment testing chemical treatments for Pd with different bats (but shared control groups) in different cages within the same incubators. Some *M. lucifugus* from the concurrent treatment experiment began to show signs of sluggishness and morbidity around day 55 (likely unrelated to WNS). Terminating the treatment experiment would have disturbed bats from the present study so it was decided to end both experiments relatively early after Pd inoculation and by 71–73 days (*M. lucifugus*) and 75–77 days (*E fuscus*) post-inoculation (mid-March) we terminated the experiment. Disease severity was assessed by qPCR^39^, UV fluorescence, and behavioral changes. Liver samples were collected, flash frozen, and shipped to Georgetown University Medical Center on dry ice for subsequent storage at -80 °C until analysis.

### Chemicals and assays used in lipidomics

Reagents for sample preparation and mass spectrometry were Optima grade (Thermo Fisher Scientific INC., Waltham, MA). SPLASH Lipidomix mass spectrometry standards were used for internal standard normalization (Avanti Polar Lipids, Inc., Alabaster, AL). Protein quantification was determined using the commercial Micro-BCA Protein Assay Kit (Thermo Fisher Scientific INC., Waltham, MA).

### Sample preparation and analysis for UPLC-QTOF-MS lipidomics

Liver samples (~ 10 mg) were homogenized with cold methanol (300 μl) containing internal standards (10 μl SPLASH Lipidomix, Avanti Polar Lipids, Inc., Alabaster, AL), 10 μl were removed for protein quantification, and the tissue homogenate was incubated on ice for 5 min. Chilled chloroform (600 μl) was added to samples, followed by shaking (vortex 30 s) and further incubation on ice (10 min). Chilled water (300 μl) was added to samples followed by shaking (vortex 30 s) and incubation on ice (10 min), then centrifuged for 10 min (10,000×*g*, 4 °C) to separate layers. The lower organic phase was separated, evaporated under vacuum, and reconstituted in 200 μl isopropanol (IPA):acetonitrile (ACN):H_2_O (2:1:1) for analysis. Five μl aliquots from each sample were combined for a quality control (QC) sample to assess instrument performance during data acquisition. A four-point standard curve of the internal standard mixture (~ 0.1–30 μg/ml) was prepared and injected before and after the experiment to assess linearity and instrument performance.

Samples were injected (2 μl) for LC separation by an ACQUITY UPLC (CSH C18 1.7 μm, 2.1 × 50 mm column) (Waters, Milford, MA) with H_2_O:ACN (1:1) + 0.1% formic acid + 10 mM ammonium formate (solvent A) and IPA:ACN (9:1) + 0.1% formic acid + 10 mM ammonium formate (solvent B) with a flow rate of 0.45 ml/min at 65 °C and gradient of 8 min 40% B, 1 min 100% B, and 2 min 40% B. Data-independent acquisition (DIA) was performed in both negative and positive electrospray ionization (ESI) with a Xevo G2-S QTOF-MS (Waters, Milford, MA). Operating conditions for ESI were: capillary voltage 2.75 kV, cone voltage 30 V, desolvation temperature 500 °C, desolvation gas flow 1000 L/Hr. Leucine enkephalin (556.2771 [M + H]^+^ or 554.2615 [M-H]^-^) was used as Lockspray to calibrate accurate mass. Ions of interest were visually inspected using tandem MS with a 5–50 V ramping collision energy.

### Data processing, statistical analysis, and marker validation

Raw chromatograms were visually inspected in MassLynx (Waters, Milford, MA), then the total ion chromatograms (TIC) were deconvoluted and peak aligned using the software Progenesis QI (Nonlinear Dynamics, Newcastle, UK). Centroid raw data files were aligned to the most suitable candidate QC file based on similarity. After manual vector alignment quality inspection, the files were aligned with sensitivity (10 ppm), retention time limits, and peak normalization (normalize to all compounds) at the default values, as previously described^40,41^. Initial putative identifications were obtained by comparing ion *m/z* to the LIPID MAPS database and MetaScope theoretical fragment search^15,42^. The dataset containing only spectral features with putative identifications was normalized to the protein concentration of each sample and respective lipid class internal standards (File S3) or normalized to all compounds in Progenesis QI for further statistical analysis. Spectral features of interest were initially identified with the software MetaboLyzer using Welch’s t test (*P* < 0.05) for ions present in at least 20% of the samples in two analysis groups or a Barnard’s test if present in less than 20% of the samples in a single group^43^. Volcano plots, principal component analysis plots (PCA, unit scaling, linear singular value decomposition, 3 maximum decision tree depth), and heatmaps were generated on complete presence ions using false discovery rates determined by Benjamini–Hochberg step-up procedure in MetaboLyzer. Lipids of interest were validated based on multiple orthogonal properties including *m/z*, adduct presence, retention time, and tandem MS spectra (File S4). Random Forests analysis was used to rank lipids between species by variable importance to model performance in MetaboAnalyst^44^. Validated ions were graphed in GraphPad Prism 6 and further assessed by an unpaired t test between sham vs. Pd-inoculated *M. lucifugus* and *E. fuscus* (GraphPad Software, Inc., La Jolla, CA). Sex differences in TG levels were assessed by a one-way ANOVA with Tukey’s multiple comparisons test (with multiple comparison corrected *P* values) and Bartlett’s test to confirm equal variances (GraphPad Software, Inc., La Jolla, CA). Effect size was determined for *M. lucifugus* and *E. fuscus* females by dividing the mean difference by the pooled standard deviation (Cohen’s *d*).

## Results

### Species differences

**Figure 1:**
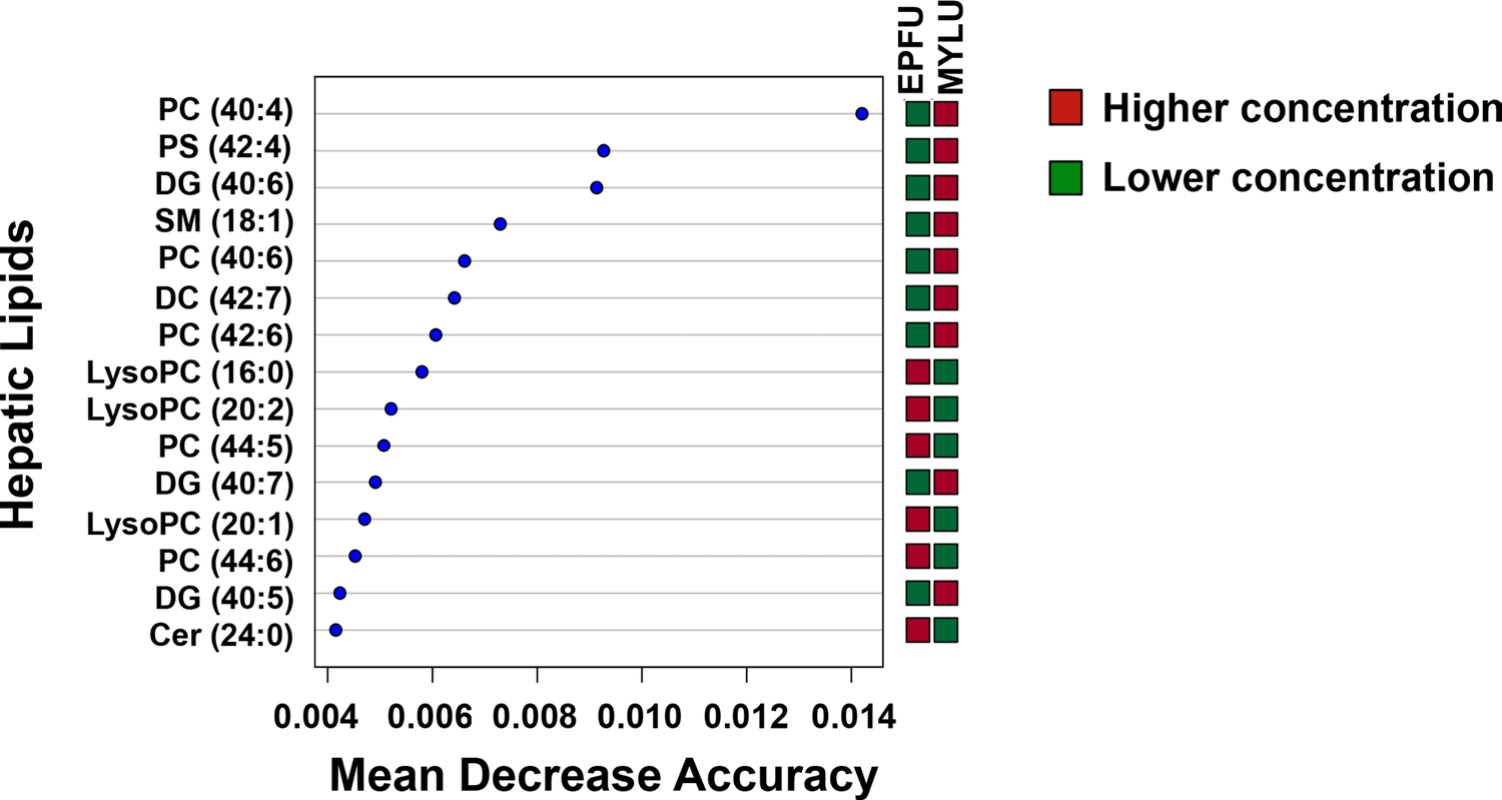
Variable importance in the Random Forests analysis of hepatic lipid concentrations between hibernating *Myotis lucifugus* (MYLU) and *Eptesicus fuscus* (EPFU). *SM* sphingomyelin, *DG* diacylglyceride, *Cer* ceramide.

A list of 214 common lipids were compiled after analyzing statistical significance of spectral features and filtering by putative LIPID MAPS identification, retention time, and observed adduct (File S3). Differences in sham treatment lipid levels between hibernating *M. lucifugus* (n = 12) and *E. fuscus* (n = 8) were assessed using a Random Forests analysis. The Random Forests machine learning algorithm is utilized extensively in the -omics field due to its high classification performance and variable selection with large spectral databases. Lipids were categorized along an x-axis by partial least squares variable importance in projection score. Higher concentrations of PC (40:4), PS (42:4), and diacylglyceride (40:6) in *M. lucifugus* and higher concentrations of LysoPCs (16:0), (20:1), (20:2), PC (44:6), and ceramide (24:0) in *E. fuscus* were identified as the top variable lipids between species (Fig. 1).

### Sex and Pd treatment differences

**Figure 2:**
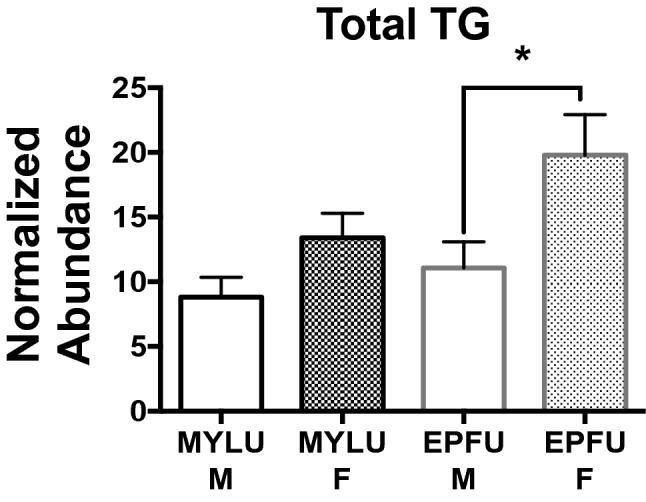
Total (> 1%) hepatic TGs in sham-inoculated *Myotis lucifugus* and *Eptesicus fuscus*. *Eptesicus fuscus* females had significantly higher concentrations of TGs compared to males. *Myotis lucifugus* females showed a moderate effect size for higher TG levels than males but not statistically significant. Mean ± S.E.M., *P ≤ 0.05, determined by a Welch’s t test, *M. lucifugus* (MYLU) n = 6 per group, *E. fuscus* (EPFU) n = 4 per group.

To determine possible sex differences in sham treatment lipid levels, we compared lipid levels between male and female *M. lucifugus* (♂ n = 6, ♀ n = 6) and *E. fuscus* (♂ n = 4, ♀ n = 4) in MetaboLyzer (Welch’s t-test). Significant differences were detected in TG concentrations between male and female sham-inoculated bats, represented in Fig. 2 as total hepatic TG (average of all TGs over > 1% of total). Total TG levels were higher for hibernating *E. fuscus* females (*P* = 0.026, Cohen's *d* = 0.7) compared to males, with a similar effect size for *M. lucifugus* females (*P* = 0.066, Cohen's *d* = 0.6) compared to males (Fig. 2).

**Figure 3:**
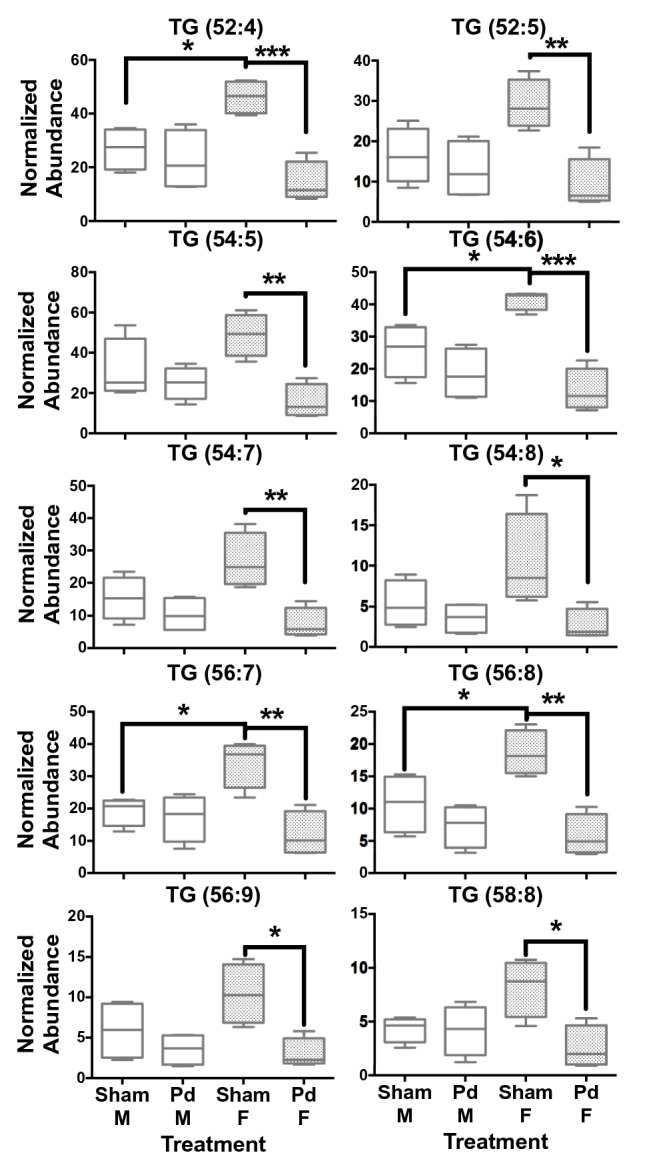
Box and whisker plots of hepatic TG concentrations in *Eptesicus fuscus*. TG concentrations tend to be highest in hibernating females compared to males. TG levels decrease significantly in females with *Pseudogymnoascus destructans* (Pd) infection compared to the sham treatment. There are no significant differences in hepatic TG concentrations between males and females in Pd-infected *E. fuscus*. *P ≤ 0.05; **P ≤ 0.01; ***P ≤ 0.001 determined by a one-way ANOVA with multiple comparisons test, lines from top to bottom represent max value, 75th percentile, median, 25th percentile, min value, n = 4 for all groups.

**Figure 4:**
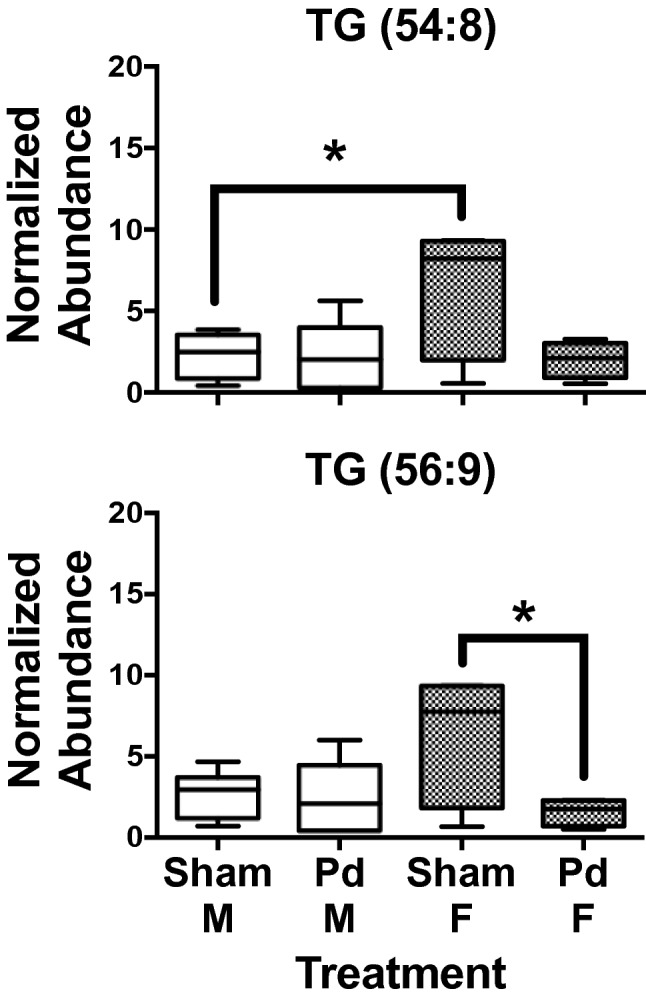
Box and whisker plots of hepatic TG concentrations in *Myotis lucifugus*. As observed in *Eptesicus fuscus*, TG concentrations tend to be higher in hibernating females compared to males. TG levels tend to decrease in females with *Pseudogymnoascus destructans* (Pd) infection compared to the sham treatment with a high effect size observed in TG (56:9) (54:8) and (56:7). *P ≤ 0.05 determined by a one-way ANOVA with multiple comparisons test, lines from top to bottom represent max value, 75th percentile, median, 25th percentile, min value, n = 6 except Pd-infected *M. lucifugus* ♀ n = 4.

Individual highly unsaturated TGs were significantly higher in female *E. fuscus* compared to males in the sham treatment for TG (52:4), (54:6), (56:7), and (56:8) (Fig. 3, File S4). Hepatic TG concentrations in Pd-inoculated female *E. fuscus* were significantly lower (0.3-fold) compared to the sham treatment (Fig. 3, Table 1). *Myotis lucifugus* females had significantly higher levels of TG (54:8) compared to males in the sham treatment (Fig. 4). Significantly lower concentrations of TG (56:9) were observed in Pd-inoculated female *M. lucifugus* and a high effect size was observed for TG (54:8) (Fig. 4, Table 1).

**Table 1:** Hepatic TG significantly altered in the white-nose syndrome (WNS)-susceptible *Myotis lucifugus* and less susceptible *Eptesicus fuscus* (sexes separate) after laboratory infection with the causal agent for WNS, *Pseudogymnoascus destructans* (Pd).

TG	*Myotis lucifugus*				*Eptesicus fuscus*			
	*P* value	FC	FC	Cohen's *d*	*P* value	FC	FC	Cohen's *d*
		Male	Female	Female		Male	Female	Female
TG (52:4)	NS	–	–	–	0.001	0.8	0.3	4.5
TG (52:5)	NS	–	–	–	0.006	0.8	0.3	3.2
TG (54:5)	NS	–	–	–	0.007	0.8	0.3	3.5
TG (54:6)	NS	–	–	–	< 0.001	0.7	0.3	5.5
TG (54:7)	NS	–	–	–	0.006	0.7	0.3	2.8
TG (54:8)	0.022	1.0	0.3	1.5	0.038	0.7	0.3	1.8
TG (56:7)	NS	–	–	–	0.003	0.9	0.3	3.1
TG (56:8)	NS	–	–	–	0.002	0.7	0.3	3.8
TG (56:9)	0.030	0.9	0.3	1.7	0.015	0.6	0.3	2.5
TG (58:8)	NS	–	–	–	0.016	1.0	0.3	2.4

FC = fold change, NS = nonsignificant, *E. fuscus* n = 4 for each sex and treatment, *M. lucifugus* n = 6 except Pd-inoculated ♀ n = 4.

**Figure 5:**
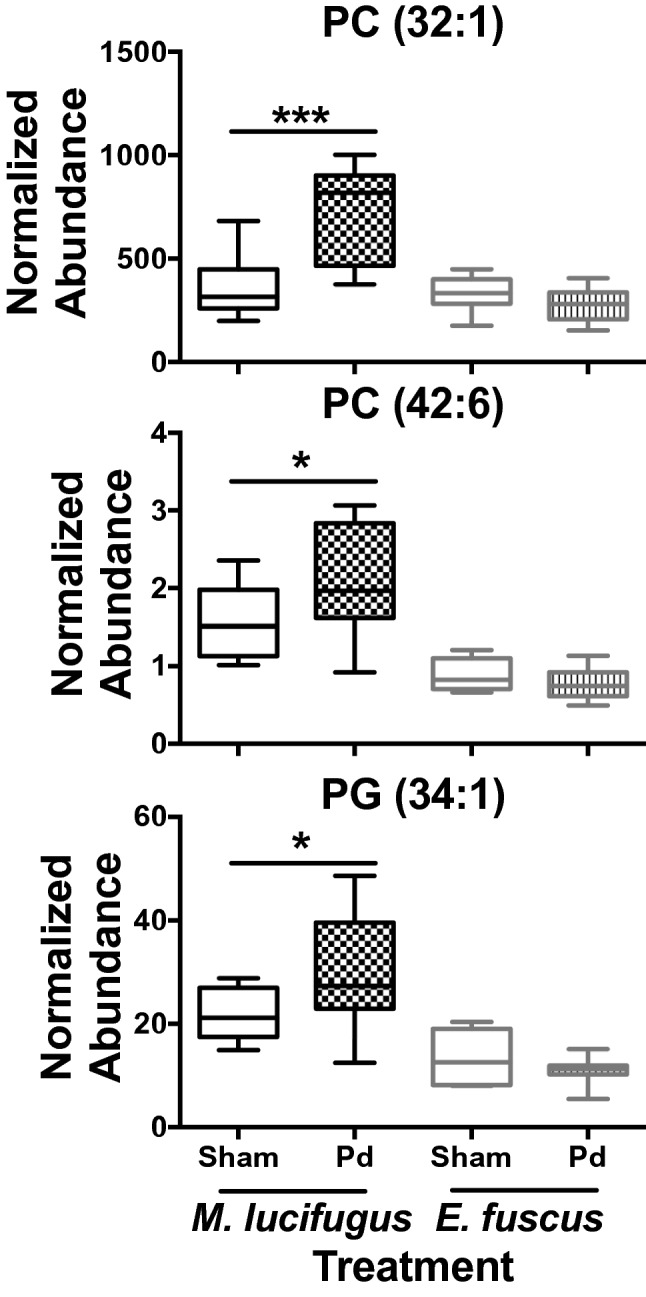
Box and whisker plots of hepatic phosphatidylcholine (PC) and phosphatidylglycerol (PG) found at higher concentrations in *Myotis lucifugus* after *Pseudogymnoascus destructans* (Pd) infection. Relative levels found in *Eptesicus fuscus* are shown as a comparison. *P ≤ 0.05; ***P ≤ 0.001 determined by an unpaired t test, lines from top to bottom represent max value, 75th percentile, median, 25th percentile, min value, *E. fuscus* n = 8 for both groups, *M. lucifugus* sham-inoculated n = 12, Pd-inoculated n = 10.

Significant changes in GP levels were only observed between sham and Pd-inoculated *M. lucifugus* and were not sex specific. *Myotis lucifugus* specific increases in hepatic GP levels compared to the sham treatment included PC (32:1) (*P* < 0.001**,** 2.0-fold), PC (42:6) (*P* = 0.04**,** 1.4-fold), and PG (34:1) (*P* = 0.041**,** 1.4-fold), which were not observed in *E. fuscus* (PC [32:1] [*P* = 0.2**,** 0.8-fold], PC [42:6] [*P* = 0.295**,** 0.9-fold], and PG [34:1) [*P* = 0.285**,** 0.8-fold]) (Fig. 5).

## Discussion

In this study, we examined changes in the bat hepatic lipidome during an early stage of Pd infection between species highly susceptible (*M. lucifugus*) or less susceptible (*E. fuscus*) to WNS and analyzed sex-specific differences. Previous studies on this cohort found Pd-inoculation destabilized *E. fuscus* skin microbiota while species that inhibit Pd growth (*Rhodococcus* and *Pseudomonas*) persisted through Pd infection^36^. While Pd transcriptomic response was similar between lesions found on both species, *E. fuscus* mounted a more localized response in wing tissue compared to *M. lucifugus*^45^. Here, we found a moderate effect size for higher hepatic TG concentrations (energy storage lipids) in hibernating females compared to males. Interestingly, we found that at early stages of Pd infection (relative to initial fungal infection), dominant TGs are significantly reduced in females but concentrations in males remained unchanged from levels observed in the sham-inoculated treatment. While differences in other lipid levels were less striking, we did see an increase in hepatic GPs (PC [32:1], PC [42:6], and PG [34:1]) in *M. lucifugus*. Overall, these results suggest that changes in lipid metabolism are occurring even at early stages of WNS and may have important implications for species and sex differences in survival and recovery.

### Glycerolipid (GL) metabolism

While a critical process during hibernation involves decreasing metabolic rate and shifting from carbohydrate to lipid based metabolism for fuel use in cursorial mammals^46,47^, bats rely more heavily on lipids as a fuel source^48^. The liver has the second highest capacity of lipid droplet storage behind adipose tissue and is the primary “hub” of lipid metabolism and fatty acid distribution. Hepatic TGs are obtained exogenously through diet or endogenously synthesized through the glycerol-3-phosphate (G3P) pathway for storage^49^. During torpor, increases in hepatic lipid metabolic gene expression (e.g., *FABP1*, *UCP2*, *ACOT12*, *ACOX1*, *EHHADH*, and *SLC27A6*)^50,51^ and hydrolysis of hepatic TGs are observed^52^. Additionally, stored white adipose TGs are hydrolyzed to diacylglycerides (patatin-like phospholipase domain-containing protein 3 [PNPLA3, EC:3.1.1.3]), MGs (hormone sensitive lipase [EC:3.1.1.79]), and eventually glycerol and free fatty acids (monoacylglycerol lipase [EC:3.1.1.23]) to be released into the bloodstream. The released fatty acids in blood can then be transported to the liver and metabolized in the mitochondria to ketone bodies through fatty acid β-oxidation or repackaged as VLDL for transportation to other tissues^53^.

Several studies have documented the association between WNS mortality and decreased fat stores^11,34,35,54^. Early observations in free-ranging bats have shown significantly decreased body fat levels in WNS-positive *M. lucifugus* compared to WNS-negative bats collected in February 2007–2008^54^. The less WNS susceptible *E. fuscus* have significantly higher mean body fat levels compared to *M. lucifugus* during mid-hibernation and spring emergence^11^. Longer-term studies have indicated that some remnant populations of *M. lucifugus* display longer torpor bout lengths, and thus, greater energy savings, and retain higher mean body fat levels over the winter, ultimately reducing WNS-associated mortality^34,35^. While these studies use estimates of total adipose tissue as a proxy for body condition, hepatic lipid composition may provide a more detailed view of fatty acid “shuttling”. Similar hepatic TG profiles are observed between male and female *M. lucifugus* and *E. fuscus*, however, we found higher concentrations of some dominant TGs in females compared to males (significantly so in *E. fuscus*). Female bats enter hibernation with higher fat reserves and are hypothesized to conserve these resources through winter compared to males to increase fecundity in spring^14,55^. Significant decreases in female hepatic TG levels during early Pd infection may indicate sex-specific challenges that could influence WNS-associated survival. Previous studies have found both increased mortality in naturally Pd-infected *M. lucifugus* females^56^ and decreased mortality in a captive cohort compared to males^57^. Clearly, given the influence of sex on lipid metabolism (e.g., hormonal differences^58^), the link to WNS survival and bat conservation is an important issue deserving of more study. Further work is also needed to examine if changes in hepatic TG content is connected to changes in overall adipose or intramuscular fat stores.

### Glycerophospholipid (GP) metabolism

Interestingly, we also found *M. lucifugus* specific increases in hepatic GP levels (PC [32:1], PC [42:6], and PG [34:1]) not observed in *E. fuscus*. Hepatic PC synthesis occurs either through the CDP-choline pathway (Kennedy pathway, ~ 70% in rodents) or through PE methylation by phosphatidylethanolamine N-methyltransferase (EC:2.1.1.17)^59^. PG synthesis occurs within mitochondria from phosphatidic acid and is a key intermediate in cardiolipin synthesis^60^. While TGs are stored within lipid droplets primarily as fuel reserves, GPs are critical to maintaining structural integrity and lipid transport in the blood. Endoplasmic reticulum derived GPs form monolayers surrounding neutral lipid cores (TGs and cholesteryl esters) of lipid droplets. Continued GP synthesis, monolayer composition (e.g., ratios of PC and PE), and fatty acid remodeling between TGs and GPs are required for lipid droplet maturation and homeostasis^61^. Similarly, GPs increase solubility of lipoproteins to enter circulation and transport TGs to adipose tissue and muscle to be metabolized back to fatty acids by lipoprotein lipase (LPL [EC:3.1.1.34]). As observed in golden-mantled ground squirrels (*Spermophilus lateralis*), hepatic PC synthesis may remain steady during different seasons in contrast to other lipogenic processes^18^. Increased apolipoprotein levels have been identified in Pd-infected *M. lucifugus* in both protein (apolipoprotein A1 and serum paraoxonase/arylesterase 1 isoform X1) and gene expression (apolipoprotein C-II, III, IV) studies^62,63^. These observed increases in apolipoprotein and hepatic PC levels in *M. lucifugus* after Pd infection may indicate differential lipid transport. This process may warrant further evaluation as differences were not detected in apolipoprotein levels in European *M. myotis*^63^ or hepatic GP levels in *E. fuscus* in the current study.

## Conclusions

Several studies have implicated increased torpor arousals, premature depletion of lipid reserves, evaporative water loss, and tissue damage as important components of WNS related mortality. As such, a detailed model of lipid metabolism during disease progression is needed. The primary tissues involved in lipid metabolism during hibernation include brown and white adipose tissues and the liver. Previous studies have documented decreases in total adipose tissue during WNS, however, the liver is a primary organ for fatty acid metabolism, lipid homeostasis, and detoxification and may provide details beyond depletion of adipose reserves. Here, we utilized a lipidomic approach to compare changes in hepatic lipid profiles in bats at an early stage of Pd infection. We observed changes in lipid profiles suggesting perturbation in lipid metabolism may be occurring early in the WNS process before significant losses in depot lipid levels. Female bats exhibited more extreme decreases in hepatic TG concentrations compared to males indicating possible sex differences in the negative consequences of WNS. These TGs also show high levels of double bonds, which should be further investigated given the importance of polyunsaturated fatty acids in torpor^64^. However, as decreases in TG concentrations were observed in species both highly (*M. lucifugus*) and less susceptible (*E. fuscus*) to WNS its role in disease dynamics remains to be elucidated. Changes in hepatic GP concentration was only observed in *M. lucifugus*, which may indicate differential lipid mobilization compared to *E. fuscus* and increased susceptibility to WNS. Future work should examine lipid composition in WNS a longitudinal manner to establish a progression of change, incorporate changes in adipose tissues and intramuscular fat stores, and employ more targeted assays to incorporate changes in bioactive lipids in addition to the structural and storage lipids examined here.

## Supplementary information

Supplementary Information.

## Data Availability

These mass spectrometry data have been deposited to the NIH data repository via Metabolomics Workbench with data set identifier ST001387.

## Abbreviations

WNS: White-nose syndrome
Pd: *Pseudogymnoascus destructans*
MYLU: *Myotis lucifugus*
EPFU: *Eptesicus fuscus*
GL: Glycerolipid
FA: Fatty acid
TG: Triacylglyceride
Cer: Ceramide
GP: Glycerophospholipid
PC: Phosphatidylcholine
LysoPC: Lysophosphatidylcholine
PG: Phosphatidylglycerol
PS: Phosphatidylserine
VLDL: Very low-density lipoprotein
UPLC: Ultra performance liquid chromatography
QTOF: Quadrupole time-of-flight
MS: Mass spectrometry
*m/z*: Mass to charge
ESI: Electrospray ionization
TIC: Total ion chromatogram
QC: Quality control
