# Supplementary material for: Hepatic lipid signatures of little brown bats (Myotis lucifugus) and big brown bats (Eptesicus fuscus) at early stages of white-nose syndrome
Source: Sci Rep. 2021 Jun 2;11:11581. doi: 10.1038/s41598-021-90828-w (PMC8172879; doi:10.1038/s41598-021-90828-w)

Hepatic lipid signatures of little brown bats (*Myotis lucifugus*) and big brown bats (*Eptesicus fuscus*) at early stages of white-nose syndrome

Evan L. Pannkuk[1,*], Nicole A. S.-Y. Dorville[2], Yvonne A. Dzal[2], Quinn E. Fletcher[2], Kaleigh J. O. Norquay[2], Craig K. R. Willis[2,*], Albert J. Fornace Jr. [1,3], Evagelia C. Laiakis[1,3]

[1]Department of Oncology, Lombardi Comprehensive Cancer Center, Georgetown University Medical Center, Washington, DC 20057, USA. [2]Department of Biology and Centre for Forest Interdisciplinary Research (C-FIR), University of Winnipeg, Winnipeg, MB, CA. [3]Department of Biochemistry and Molecular & Cellular Biology, Georgetown University Medical Center, Washington, DC 20057, USA. [*]Correspondence and request for materials should be addressed to E.L.P. (elp44@georgetown.edu) or C.K.R.W. (c.willis@uwinnipeg.ca)

File S1. 1- Ct values from qPCR and wing fluorescence values.

bat.id	infection type	pre-treatment Ct-value	post-treatment Ct-value	orange.uv.proportion
IE_2017_MYLU_115	PBST	NA	undetermined	0
IE_2017_MYLU_34	PBST	NA	undetermined	0
IE_2017_MYLU_38	PBST	NA	undetermined	0
IE_2017_MYLU_55	PBST	undetermined	undetermined	0
IE_2017_MYLU_6	PBST	NA	undetermined	0
IE_2017_MYLU_65	PBST	NA	undetermined	0
IE_2017_MYLU_78	PBST	NA	undetermined	0
IE_2017_MYLU_83	PBST	NA	39.95958176	0
IE_2017_MYLU_84	PBST	undetermined	undetermined	0
IE_2017_MYLU_89	PBST	NA	undetermined	0
IE_2017_MYLU_9	PBST	NA	undetermined	0
IE_2017_MYLU_95	PBST	undetermined	undetermined	0
IE_2017_MYLU_21	Pd	NA	31.92546806	0.321
IE_2017_MYLU_70	Pd	NA	30.28710079	0.063
IE_2017_MYLU_73	Pd	NA	34.80744553	0.53
IE_2017_MYLU_76	Pd	NA	33.52658463	0.147
IE_2017_MYLU_85	Pd	NA	32.94957924	4.684
IE_2017_MYLU_81	Pd	undetermined	31.01336002	5.602
IE_2017_MYLU_1	Pd	NA	35.74436378	0.181
IE_2017_MYLU_15	Pd	NA	31.89715443	0.45
IE_2017_MYLU_28	Pd	NA	31.36805725	0.457
IE_2017_MYLU_109	Pd	NA	30.70916367	0.515
IE_2017_EPFU_13	PBST	undetermined	undetermined	0
IE_2017_EPFU_15	PBST	undetermined	37.04833603	0
IE_2017_EPFU_19	PBST	undetermined	undetermined	0
IE_2017_EPFU_24	PBST	undetermined	undetermined	0
IE_2017_EPFU_28	PBST	NA	39.66645432	0
IE_2017_EPFU_31	PBST	39.55136335	undetermined	0.077
IE_2017_EPFU_7	PBST	undetermined	39.70586777	0.01
IE_2017_EPFU_9	PBST	39.49631691	undetermined	0.021
IE_2017_EPFU_10	Pd	39.52378273	32.56645966	6.758
IE_2017_EPFU_16	Pd	undetermined	35.51846504	0.203
IE_2017_EPFU_20	Pd	undetermined	34.37360573	0.731
IE_2017_EPFU_23	Pd	39.36528415	34.9600029	1.35
IE_2017_EPFU_27	Pd	undetermined	38.65805817	0.517
IE_2017_EPFU_8	Pd	39.11196223	35.44903374	0.074
IE_2017_EPFU_1	Pd	undetermined	33.46580124	8.416

File S2. Sample sizes for treatment groups.

species	sex	treatment	n
MYLU	M	sham	6
MYLU	F	sham	6
MYLU	M	Pd	6
MYLU	F	Pd	4
EPFU	M	sham	4
EPFU	F	sham	4
EPFU	M	Pd	4
EPFU	F	Pd	4

File S3. Table listing *m/z*, retention times, formula, and measured adducts of common lipids identified in this study. Normalized abundance values are given between *Myotis lucifugus* and *Eptesicus fuscus*.

m/z_rt	Lipid Species	Adducts	Formula	*E. fuscus*	*M. lucifugus*
.	.	.	.	n=8	n=12
379.28_2.15	MG(18:1)	M+Na	C21H40O4	0.028	0.083
377.27_1.66	MG(18:2)	M+Na	C21H38O4	0.050	0.096
403.28_1.48	MG(22:6)	M+Na	C25H38O4	0.067	0.167
549.48_5.63	DG(32:1)	M+NH4	C35H66O5	1.313	2.140
582.51_5.27	DG(32:2)	M+NH4	C35H64O5	0.039	0.073
577.52_6.00	DG(34:1)	M+NH4	C37H70O5	3.745	4.375
575.50_5.70	DG(34:2)	M+NH4	C37H68O5	10.465	11.280
573.49_5.36	DG(34:3)	M+NH4	C37H66O5	2.778	3.323
589.48_5.05	DG(34:4)	M+NH4	C37H64O5	0.817	1.319
605.55_6.33*	DG(36:1)	M+NH4	C39H74O5	0.698	1.814
603.53_6.03*	DG(36:2)	M+NH4	C39H72O5	10.671	17.833
641.51_5.75	DG(36:3)	M+NH4	C39H70O5	16.074	21.994
617.51_5.45	DG(36:4)	M+NH4	C39H68O5	13.597	16.743
632.52_5.15	DG(36:5)	M+NH4	C39H66O5	1.195	1.696
630.51_4.83	DG(36:6)	M+NH4	C39H64O5	0.100	0.135
631.56_6.33	DG(38:2)	M+NH4	C41H76O5	0.149	0.333
662.57_6.02	DG(38:4)	M+NH4	C41H72O5	6.803	7.032
660.56_5.67	DG(38:5)	M+NH4	C41H70O5	8.124	9.472
658.54_5.36	DG(38:6)	M+NH4	C41H68O5	2.642	3.220
688.58_6.03*	DG(40:5)	M+NH4	C43H74O5	0.629	1.577
686.57_5.70*	DG(40:6)	M+NH4	C43H72O5	3.987	8.555
684.55_5.41*	DG(40:7)	M+NH4	C43H70O5	1.462	3.225
714.60_5.99*	DG(42:6)	M+NH4	C45H76O5	0.055	0.457
712.58_5.72	DG(42:7)	M+NH4	C45H74O5	0.071	0.374
610.55_5.89 int std	DG(15:0/18:1)d7	M+Na	C36H61D7O5		
255.23_2.65	FFA(16:0)	M-H	C16H32O2	0.910	1.137
253.22_2.02	FFA(16:1)	M-H	C16H30O2	0.298	0.619
283.26_3.43	FFA(18:0)	M-H	C18H36O2	1.558	2.129
281.25_2.76	FFA(18:1)	M-H	C18H34O2	2.910	4.313
279.23_2.21	FFA(18:2)	M-H	C18H32O2	2.741	4.061
277.22_1.75	FFA(18:3)	M-H	C18H30O2	0.839	1.101
301.22_1.67	FFA(20:3)	M-H	C20H30O2	1.122	1.827
303.23_2.11	FFA(20:4)	M-H	C20H32O2	6.884	5.444
329.25_2.23*	FFA(22:5)	M-H	C22H34O2	1.251	2.996
327.23_1.97	FFA(22:6)	M-H	C22H32O2	1.740	1.269
468.31_0.88	LysoPC(14:0)	M+H	C22H46NO7P	1.515	0.855
496.34_1.19*	LysoPC(16:0)	M+H	C24H50NO7P	146.894	89.835
494.32_0.94	LysoPC(16:1)	M+H	C24H48NO7P	15.329	7.244
524.37_1.73	LysoPC(18:0)	M+H	C26H54NO7P	43.768	56.779
522.36_1.29	LysoPC(18:1)	M+H	C26H52NO7P	64.248	32.816

520.34_0.95	LysoPC(18:2)	M+H	C26H50NO7P	53.321	18.252
550.38_1.82	LysoPC(20:1)	M+H	C28H56NO7P	0.381	0.228
548.37_1.39	LysoPC(20:2)	M+H	C28H54NO7P	0.208	0.053
544.34_0.94	LysoPC(20:4)	M+H	C28H50NO7P	19.410	5.647
568.34_0.91	LysoPC(22:6)	M+H	C30H50NO7P	7.406	1.512
529.40_1.34 int std	LysoPC(18:1)d7	M+H	C26H45D7NO7P		
452.28_1.27	LysoPE(16:0)	M-H	C21H44NO7P	0.822	0.495
450.26_0.98	LysoPE(16:1)	M-H	C21H42NO7P	0.102	0.073
480.31_1.84	LysoPE(18:0)	M-H	C23H48NO7P	2.100	2.107
478.29_1.36	LysoPE(18:1)	M-H	C23H46NO7P	4.359	2.832
476.28_1.01	LysoPE(18:2)	M-H	C23H44NO7P	1.767	0.668
474.26_0.86	LysoPE(18:3)	M-H	C23H42NO7P	0.222	0.072
485.33_1.42 int std	LysoPE(18:1)d7	M-H	C23H39D7NO7P		
706.54_4.60	PC(30:0)	M+H	C38H76NO8P	8.151	6.096
734.57_5.06*	PC(32:0)	M+H	C40H80NO8P	54.469	29.559
732.55_4.69	PC(32:1)	M+H	C40H78NO8P	328.488	370.097
728.52_3.92	PC(32:3)	M+H	C40H74NO8P	29.819	29.733
726.50_3.66	PC(32:4)	M+H	C40H72NO8P	2.543	2.254
724.49_3.26	PC(32:5)	M+H	C40H70NO8P	0.575	0.417
760.59_5.14	PC(34:1)	M+H	C42H82NO8P	1396.609	1136.960
758.57_4.80	PC(34:2)	M+H	C42H80NO8P	1695.445	1695.889
754.54_4.22	PC(34:4)	M+H	C42H76NO8P	56.073	42.868
752.52_3.88	PC(34:5)	M+H	C42H74NO8P	12.692	12.235
788.62_5.55	PC(36:1)	M+H	C44H86NO8P	325.358	341.038
786.60_5.24	PC(36:2)	M+H	C44H84NO8P	677.451	957.592
782.57_4.53	PC(36:4)	M+H	C44H80NO8P	670.291	699.494
778.54_3.78	PC(36:6)	M+H	C44H76NO8P	63.083	40.444
816.64_5.90	PC(38:1)	M+H	C46H90NO8P	5.753	3.488
812.61_5.36*	PC(38:3)	M+H	C46H86NO8P	21.825	37.752
810.60_5.18	PC(38:4)	M+H	C46H84NO8P	271.122	367.000
802.54_3.73	PC(38:8)	M+H	C46H76NO8P	14.230	12.909
800.52_3.41	PC(38:9)	M+H	C46H74NO8P	0.566	0.450
844.68_6.23	PC(40:1)	M+H	C48H94NO8P	0.768	0.340
842.66_5.90	PC(40:2)	M+H	C48H92NO8P	4.312	2.445
840.64_5.65	PC(40:3)	M+H	C48H90NO8P	3.655	3.143
838.63_5.48*	PC(40:4)	M+H	C48H88NO8P	8.842	15.939
834.60_4.83*	PC(40:6)	M+H	C48H84NO8P	30.673	52.966
830.57_4.13	PC(40:8)	M+H	C48H80NO8P	15.464	14.421
872.71_6.53*	PC(42:1)	M+H	C50H98NO8P	0.418	0.114
854.57_4.24	PC(42:10)	M+H	C50H80NO8P	8.673	9.386
852.55_3.89	PC(42:11)	M+H	C50H78NO8P	0.567	0.533
870.69_6.21*	PC(42:2)	M+H	C50H96NO8P	0.710	0.289

868.67_5.99	PC(42:3)	M+H	C50H94NO8P	0.563	0.299
864.64_5.59	PC(42:5)	M+H	C50H90NO8P	3.515	3.712
862.63_5.16*	PC(42:6)	M+H	C50H88NO8P	0.885	1.559
860.61_4.88	PC(42:7)	M+H	C50H86NO8P	0.758	1.128
858.60_4.60	PC(42:8)	M+H	C50H84NO8P	0.835	1.199
894.69_6.26	PC(44:4)	M+H	C52H96NO8P	0.125	0.035
892.68_5.93*	PC(44:5)	M+H	C52H94NO8P	0.307	0.087
890.66_5.60*	PC(44:6)	M+H	C52H92NO8P	0.110	0.042
753.61_5.00 int std	PC(15:0/18:1)d7	M+H	C41H73D7NO8P		
688.49_4.82	PE(32:1)	M-H	C37H72NO8P	0.611	1.055
714.51_4.93	PE(34:2)	M-H	C39H74NO8P	14.437	18.659
712.49_4.53	PE(34:3)	M-H	C39H72NO8P	12.040	12.906
710.48_4.17	PE(34:4)	M-H	C39H70NO8P	1.045	1.234
744.55_5.14	PE(36:1)	M-H	C41H80NO8P	6.898	6.365
742.54_4.80	PE(36:2)	M-H	C41H78NO8P	6.140	6.631
740.52_4.39	PE(36:3)	M-H	C41H76NO8P	3.246	3.722
772.58_5.55	PE(38:1)	M-H	C43H84NO8P	1.289	1.666
770.57_5.24*	PE(38:2)	M-H	C43H82NO8P	3.919	6.103
768.55_4.86*	PE(38:3)	M-H	C43H80NO8P	3.302	4.855
760.49_4.24	PE(38:7)	M-H	C43H72NO8P	9.403	8.122
758.48_3.86	PE(38:8)	M-H	C43H70NO8P	0.860	0.696
794.57_5.59	PE(40:4)	M-H	C45H82NO8P	2.211	3.260
790.54_5.21	PE(40:6)	M-H	C45H78NO8P	28.008	36.450
788.52_4.82	PE(40:7)	M-H	C45H76NO8P	91.562	95.277
784.49_4.12	PE(40:9)	M-H	C45H72NO8P	3.449	1.902
709.55_5.01 int std	PE(15:0/18:1)d7	M-H	C38H67D7NO8P		
719.49_4.31	PG(32:1)	M-H	C38H73O10P	0.116	0.080
747.52_4.76*	PG(34:1)	M-H	C40H77O10P	13.214	22.071
745.50_4.45	PG(34:2)	M-H	C40H75O10P	22.032	25.326
743.48_4.10	PG(34:3)	M-H	C40H73O10P	0.119	0.054
773.53_4.54	PG(36:2)	M-H	C42H79O10P	15.124	10.150
771.52_4.18	PG(36:3)	M-H	C42H77O10P	6.757	4.065
769.50_3.82	PG(36:4)	M-H	C42H75O10P	2.939	2.061
767.48_3.44	PG(36:5)	M-H	C42H73O10P	1.433	1.116
795.52_4.14	PG(38:5)	M-H	C44H77O10P	3.352	2.090
793.50_3.76	PG(38:6)	M-H	C44H75O10P	1.968	1.155
791.49_3.41	PG(38:7)	M-H	C44H73O10P	1.210	0.807
819.52_4.03	PG(40:7)	M-H	C46H77O10P	3.499	1.665
817.50_3.66	PG(40:8)	M-H	C46H75O10P	1.345	0.832
815.48_3.29	PG(40:9)	M-H	C46H73O10P	0.364	0.181
841.50_3.59	PG(42:10)	M-H	C48H75O10P	0.422	0.212
839.49_3.23	PG(42:11)	M-H	C48H73O10P	0.031	0.010

865.50_3.48	PG(44:12)	M-H	C50H75O10P	0.250	0.123
740.55_4.53 int std	PG(15:0/18:1)d7	M-H	C39H68D7O10P		
786.53_4.86	PS(36:2)	M-H	C42H78NO10P	3.790	5.438
782.50_4.32*	PS(36:4)	M-H	C42H74NO10P	0.578	0.199
818.59_5.36	PS(38:0)	M-H	C44H86NO10P	6.845	6.167
816.57_5.04	PS(38:1)	M-H	C44H84NO10P	22.225	24.448
850.56_4.64	PS(38:2)	M-H	C44H82NO10P	32.779	29.938
812.54_4.28	PS(38:3)	M-H	C44H80NO10P	1.799	2.075
846.62_5.72	PS(40:0)	M-H	C46H90NO10P	2.719	1.583
844.60_5.45	PS(40:1)	M-H	C46H88NO10P	3.736	3.413
840.57_4.98	PS(40:3)	M-H	C46H84NO10P	7.216	8.120
836.54_4.82	PS(40:5)	M-H	C46H80NO10P	8.393	10.933
834.53_4.69	PS(40:6)	M-H	C46H78NO10P	10.320	6.460
868.60_5.41	PS(42:3)	M-H	C48H88NO10P	1.587	1.183
866.59_5.01*	PS(42:4)	M-H	C48H86NO10P	1.882	3.265
864.57_4.87	PS(42:5)	M-H	C48H84NO10P	1.394	1.480
862.56_4.46	PS(42:6)	M-H	C48H82NO10P	0.406	0.267
894.62_5.42	PS(44:4)	M-H	C50H90NO10P	0.099	0.151
753.54_4.46 int std	PS(15:0/18:1)d7	M-H	C39H67D7NO10P		
647.51_3.35	SM(12:0)	M+H	C35H71N2O6P	6.865	4.824
675.54_4.00	SM(14:0)	M+H	C37H75N2O6P	121.521	90.620
731.61_5.05	SM(18:0)	M+H	C41H83N2O6P	38.757	45.962
729.59_4.64*	SM(18:1)	M+H	C41H81N2O6P	7.996	20.139
759.64_5.48	SM(20:0)	M+H	C43H87N2O6P	37.230	27.402
757.62_5.11	SM(20:1)	M+H	C43H85N2O6P	16.500	18.581
787.67_5.85	SM(22:0)	M+H	C45H91N2O6P	47.851	26.283
785.65_5.55*	SM(22:1)	M+H	C45H89N2O6P	36.067	24.997
815.70_6.20	SM(24:0)	M+H	C47H95N2O6P	16.828	10.631
813.68_5.84	SM(24:1)	M+H	C47H93N2O6P	71.049	42.211
843.73_6.46	SM(26:0)	M+H	C49H99N2O6P	0.144	0.103
841.71_6.18	SM(26:1)	M+H	C49H97N2O6P	0.396	0.201
464.44_4.21	Cer(12:0)	M+H-H2O	C30H59NO3	0.272	0.117
492.48_4.75	Cer(14:0)	M+H-H2O	C32H63NO3	0.651	0.448
520.51_5.23*	Cer(16:0)	M+H-H2O	C34H67NO3	2.896	1.746
548.54_5.69	Cer(18:0)	M+H-H2O	C36H71NO3	1.477	1.185
546.52_5.30	Cer(18:1)	M+H-H2O	C36H69NO3	0.152	0.270
576.57_6.03	Cer(20:0)	M+H-H2O	C38H75NO3	0.886	0.555
604.60_6.38	Cer(22:0)	M+H-H2O	C40H79NO3	0.599	0.341
632.63_6.67	Cer(24:0)	M+H-H2O	C42H83NO3	0.289	0.118
630.61_6.35*	Cer(24:1)	M+H-H2O	C42H81NO3	1.937	0.808
738.65_4.73 int std	SM(d18:1/18:1)d9	M+H	C41H72D9N2O6P		
684.61_6.71	TG(38:0)	M+NH4	C41H78O6	0.126	0.131

712.64_6.97	TG(40:0)	M+NH4	C43H82O6	0.118	0.101
740.67_7.20	TG(42:0)	M+NH4	C45H86O6	0.108	0.111
736.64_6.79	TG(42:2)	M+NH4	C45H82O6	0.662	0.203
734.63_6.61	TG(42:3)	M+NH4	C45H80O6	0.396	0.097
768.70_7.41	TG(44:0)	M+NH4	C47H90O6	0.136	0.173
766.69_7.20	TG(44:1)	M+NH4	C47H88O6	0.266	0.365
764.67_7.00	TG(44:2)	M+NH4	C47H86O6	1.176	1.006
762.66_6.83	TG(44:3)	M+NH4	C47H84O6	1.518	0.630
760.64_6.61	TG(44:4)	M+NH4	C47H82O6	1.251	0.362
796.73_7.61	TG(46:0)	M+NH4	C49H94O6	0.249	0.278
794.72_7.43	TG(46:1)	M+NH4	C49H92O6	0.305	0.592
792.70_7.22	TG(46:2)	M+NH4	C49H90O6	1.390	1.882
790.69_7.04	TG(46:3)	M+NH4	C49H88O6	1.718	1.653
788.67_6.90	TG(46:4)	M+NH4	C49H86O6	1.999	1.228
786.66_6.74	TG(46:5)	M+NH4	C49H84O6	1.066	0.518
824.76_7.77	TG(48:0)	M+NH4	C51H98O6	0.359	0.388
822.75_7.61	TG(48:1)	M+NH4	C51H96O6	0.579	0.933
820.73_7.43	TG(48:2)	M+NH4	C51H94O6	3.029	4.317
818.72_7.25	TG(48:3)	M+NH4	C51H92O6	4.085	4.554
812.67_6.84	TG(48:6)	M+NH4	C51H86O6	1.676	1.041
810.66_6.65	TG(48:7)	M+NH4	C51H84O6	0.756	0.324
852.80_7.94	TG(50:0)	M+NH4	C53H102O6	0.165	0.191
850.78_7.77	TG(50:1)	M+NH4	C53H100O6	0.520	0.622
848.77_7.61	TG(50:2)	M+NH4	C53H98O6	5.904	6.627
846.75_7.45	TG(50:3)	M+NH4	C53H96O6	12.418	12.253
844.74_7.30	TG(50:4)	M+NH4	C53H94O6	10.176	8.630
842.72_7.12	TG(50:5)	M+NH4	C53H92O6	7.087	5.535
840.70_6.95	TG(50:6)	M+NH4	C53H90O6	3.718	2.892
836.67_6.69	TG(50:8)	M+NH4	C53H86O6	0.388	0.224
880.83_8.10	TG(52:0)	M+NH4	C55H106O6	0.107	0.133
876.80_7.79	TG(52:2)	M+NH4	C55H102O6	6.592	5.456
872.77_7.48	TG(52:4)	M+NH4	C55H98O6	36.554	24.997
870.75_7.33	TG(52:5)	M+NH4	C55H96O6	22.745	15.264
868.74_7.15	TG(52:6)	M+NH4	C55H94O6	10.062	6.593
866.72_6.98	TG(52:7)	M+NH4	C55H92O6	4.768	3.217
864.70_6.86	TG(52:8)	M+NH4	C55H90O6	1.385	0.886
904.83_7.94	TG(54:2)	M+NH4	C57H106O6	0.384	0.655
902.81_7.79	TG(54:3)	M+NH4	C57H104O6	6.726	7.329
900.80_7.64	TG(54:4)	M+NH4	C57H102O6	24.290	20.239
898.78_7.49	TG(54:5)	M+NH4	C57H100O6	39.935	26.827
896.77_7.34	TG(54:6)	M+NH4	C57H98O6	33.605	20.107
894.75_7.18	TG(54:7)	M+NH4	C57H96O6	21.045	11.677

892.73_7.01	TG(54:8)	M+NH4	C57H94O6	7.818	4.313
890.72_6.90	TG(54:9)	M+NH4	C57H92O6	2.281	1.186
916.73_6.95	TG(56:10)	M+NH4	C59H94O6	3.077	1.603
914.72_6.84	TG(56:11)	M+NH4	C59H92O6	0.286	0.173
922.78_7.43	TG(56:7)	M+NH4	C59H100O6	26.792	20.008
920.77_7.28	TG(56:8)	M+NH4	C59H98O6	14.680	9.038
918.75_7.12	TG(56:9)	M+NH4	C59H96O6	8.150	4.427
944.77_7.20	TG(58:10)	M+NH4	C61H98O6	5.456	2.884
942.75_7.04	TG(58:11)	M+NH4	C61H96O6	1.921	0.926
940.73_6.89	TG(58:12)	M+NH4	C61H94O6	0.519	0.238
952.83_7.73	TG(58:6)	M+NH4	C61H106O6	0.341	0.512
948.80_7.43	TG(58:8)	M+NH4	C61H102O6	6.260	5.575
968.76_7.13	TG(60:12)	M+NH4	C63H98O6	0.708	0.281
966.75_6.98	TG(60:13)	M+NH4	C63H96O6	0.203	0.044
829.80_7.66 int std	TG(15:0/18:1/15:0)d7	M+NH4	C51H89D7O6		
*P < 0.01 Welch's t-test *E. fuscus* vs. *M. lucifugus* sham inoculated					

File S4. Tandem MS spectra of significant lipids identified between sham vs. Pd-inoculated *Myotis lucifugus* and *Eptesicus fuscus*.

TG (52:4)
Positive Mode
MS/MS

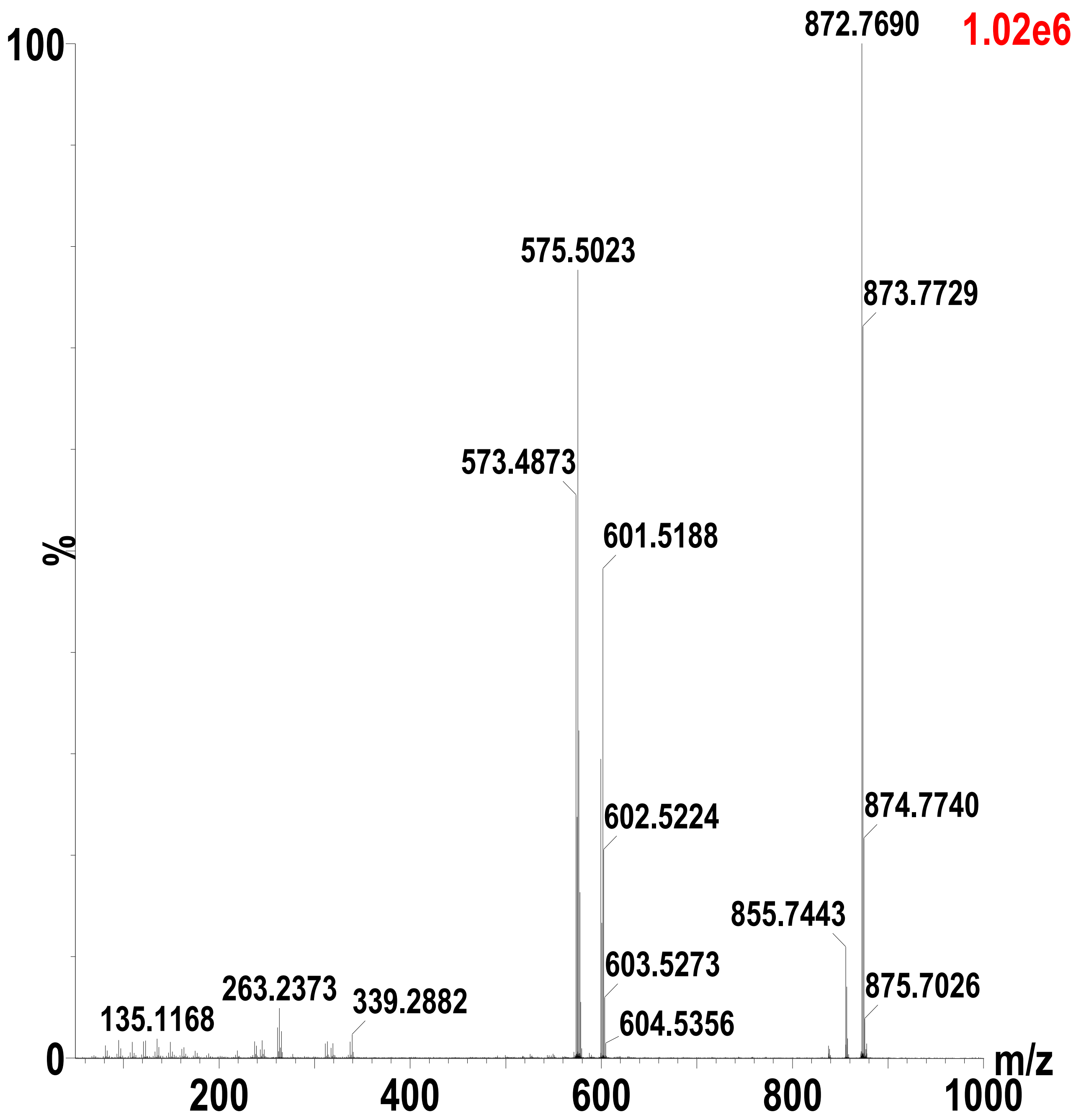

TG (52:5)
Positive Mode
MS/MS

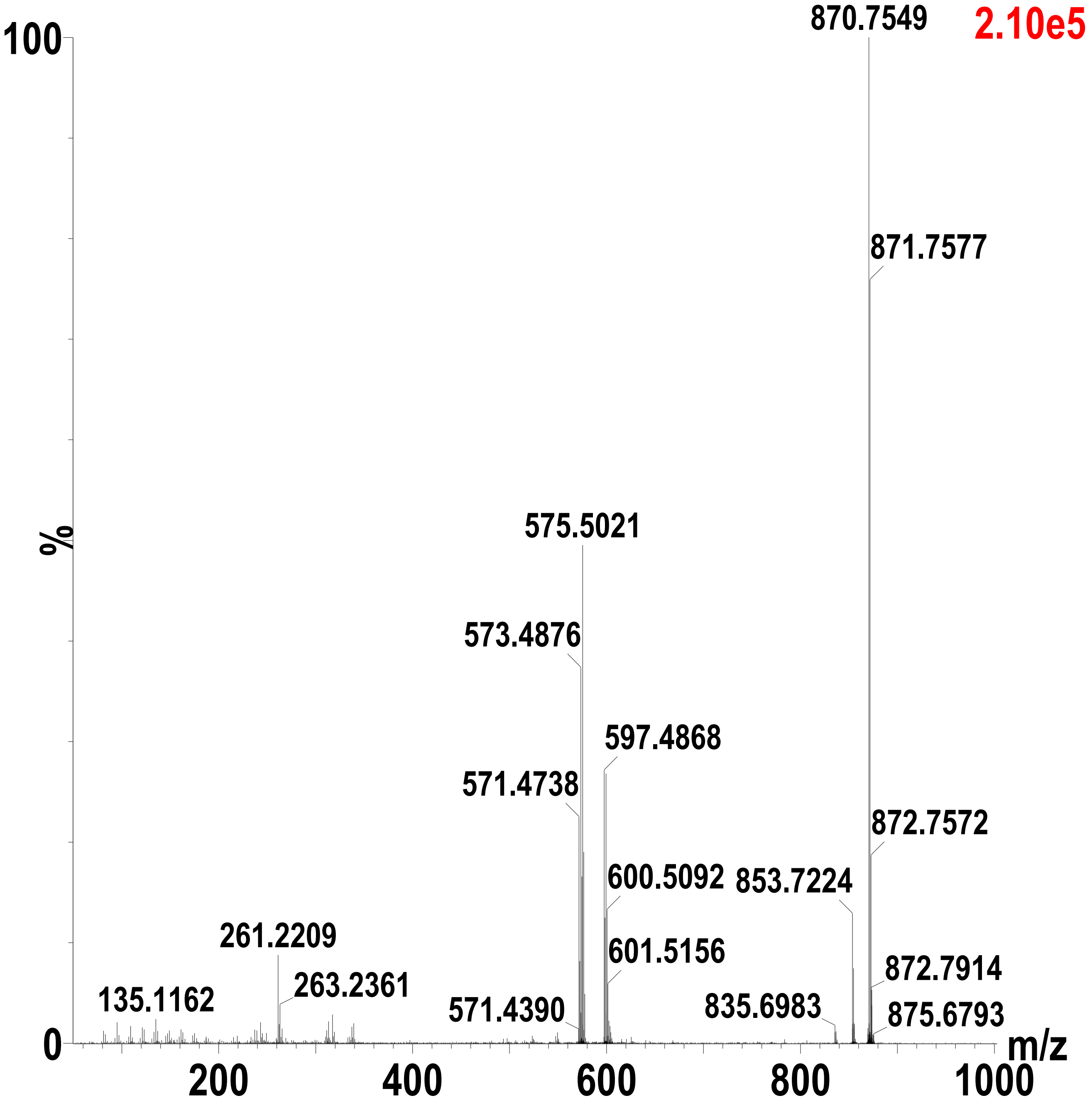

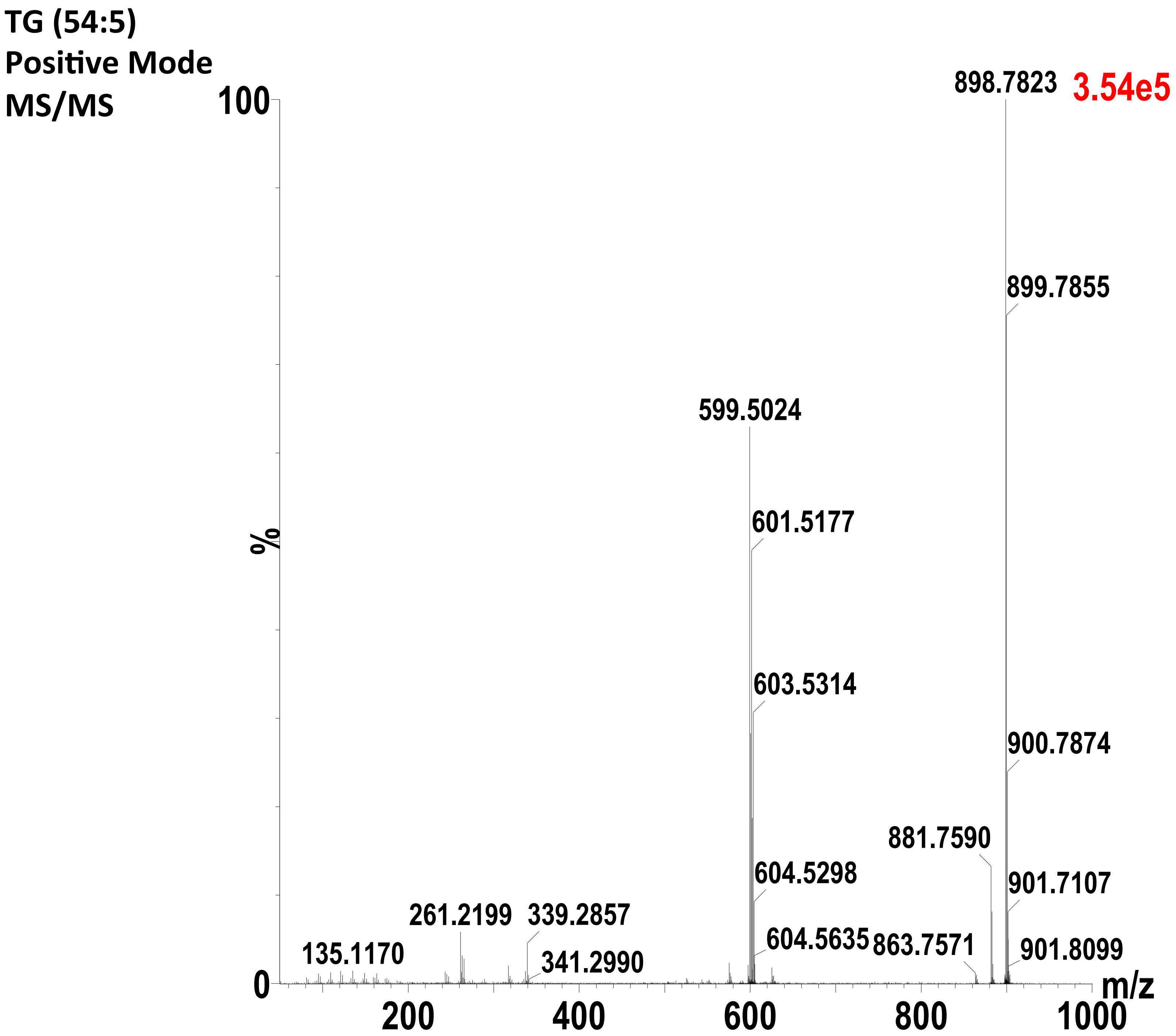

TG (54:5)
Positive Mode
MS/MS
100
898.7823
3.54e5
899.7855
599.5024
601.5177
%
603.5314
900.7874
881.7590
604.5298
901.7107
261.2199
339.2857
604.5635
863.7571
901.8099
135.1170
341.2990
0
200
400
600
800
1000
m/z

TG (54:6)
Positive Mode
MS/MS

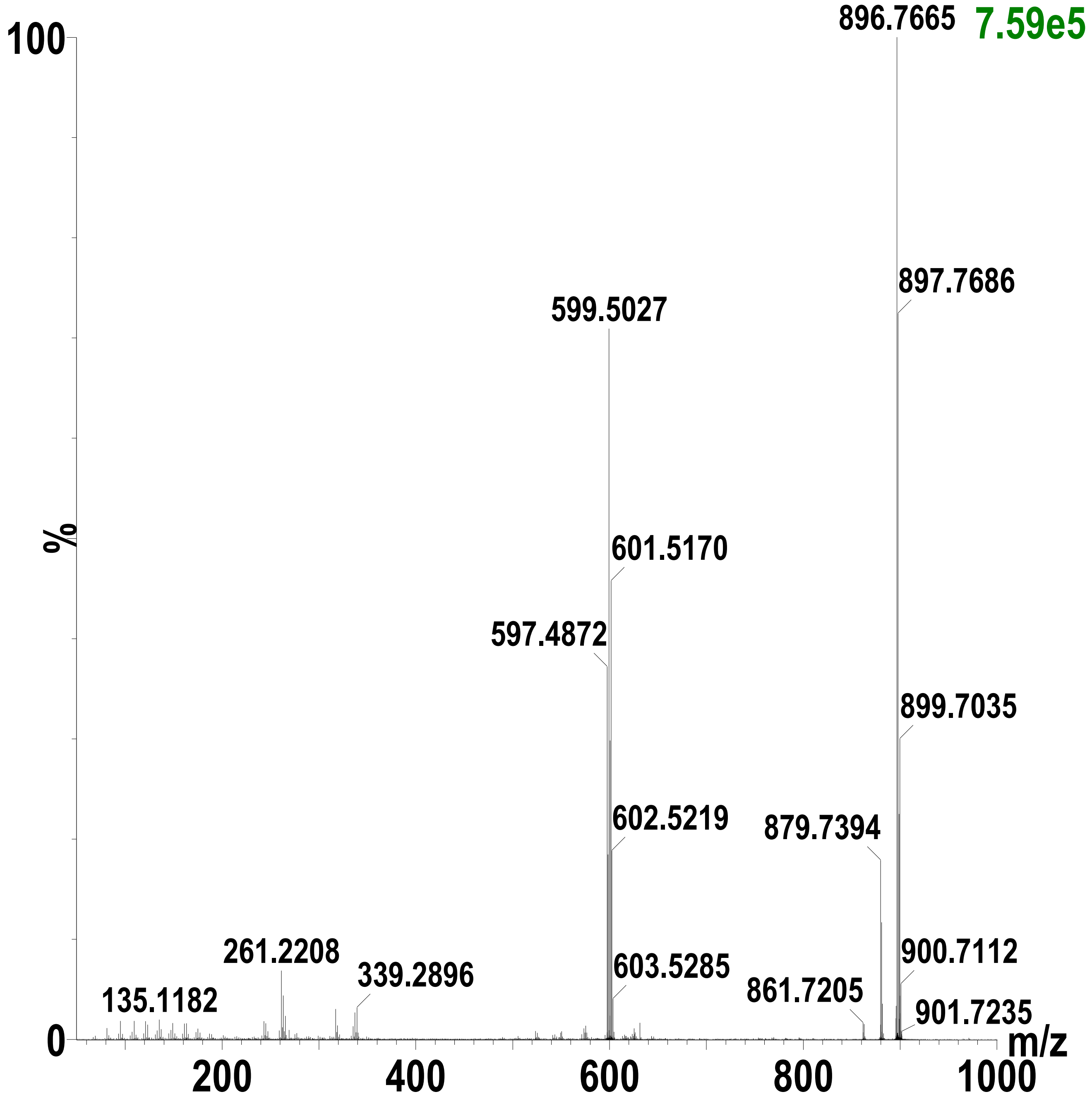

TG (54:7)
Positive Mode
MS/MS

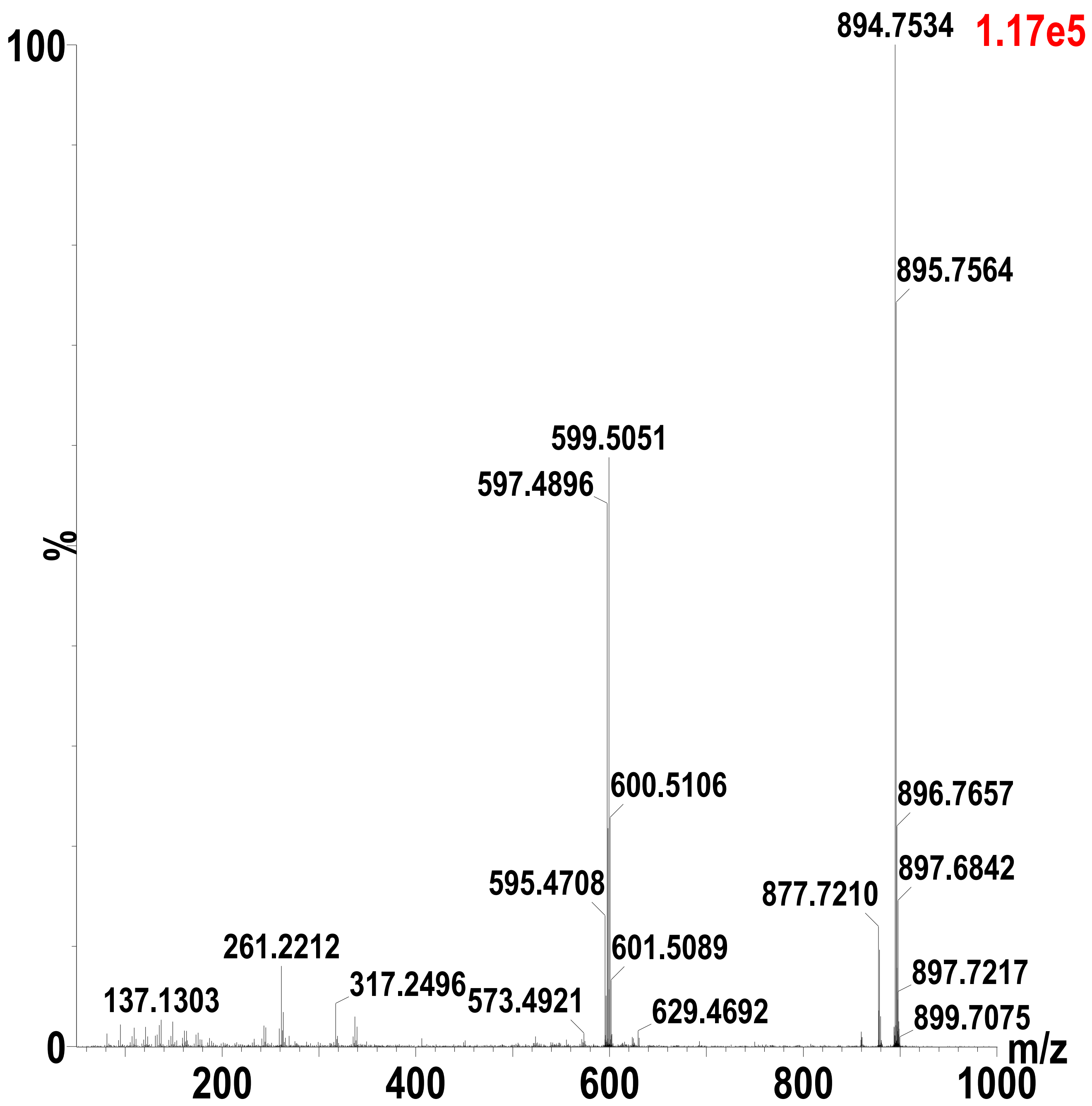

TG (54:8)
Positive Mode
MS/MS

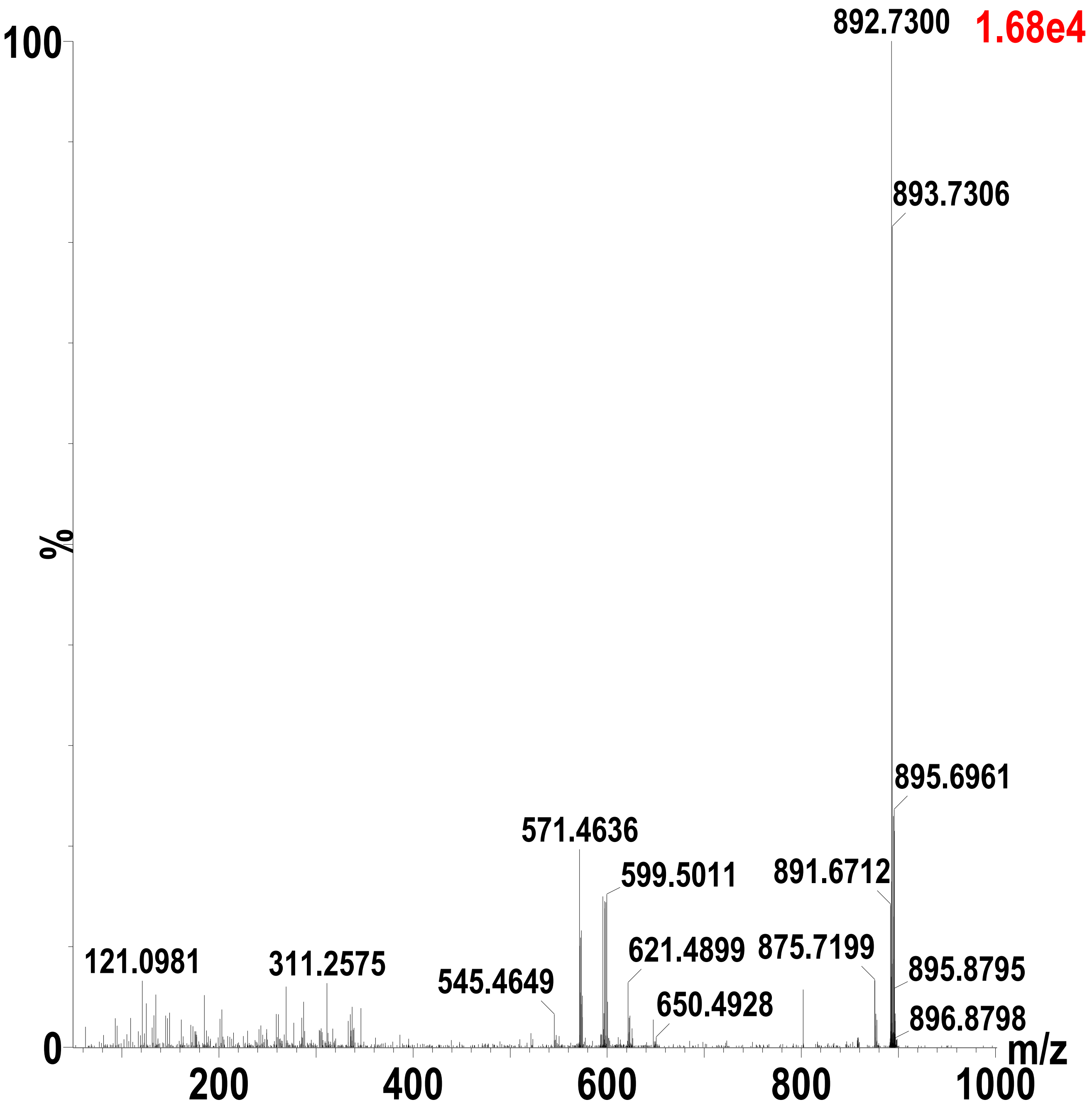

TG (56:7)
Positive Mode
MS/MS

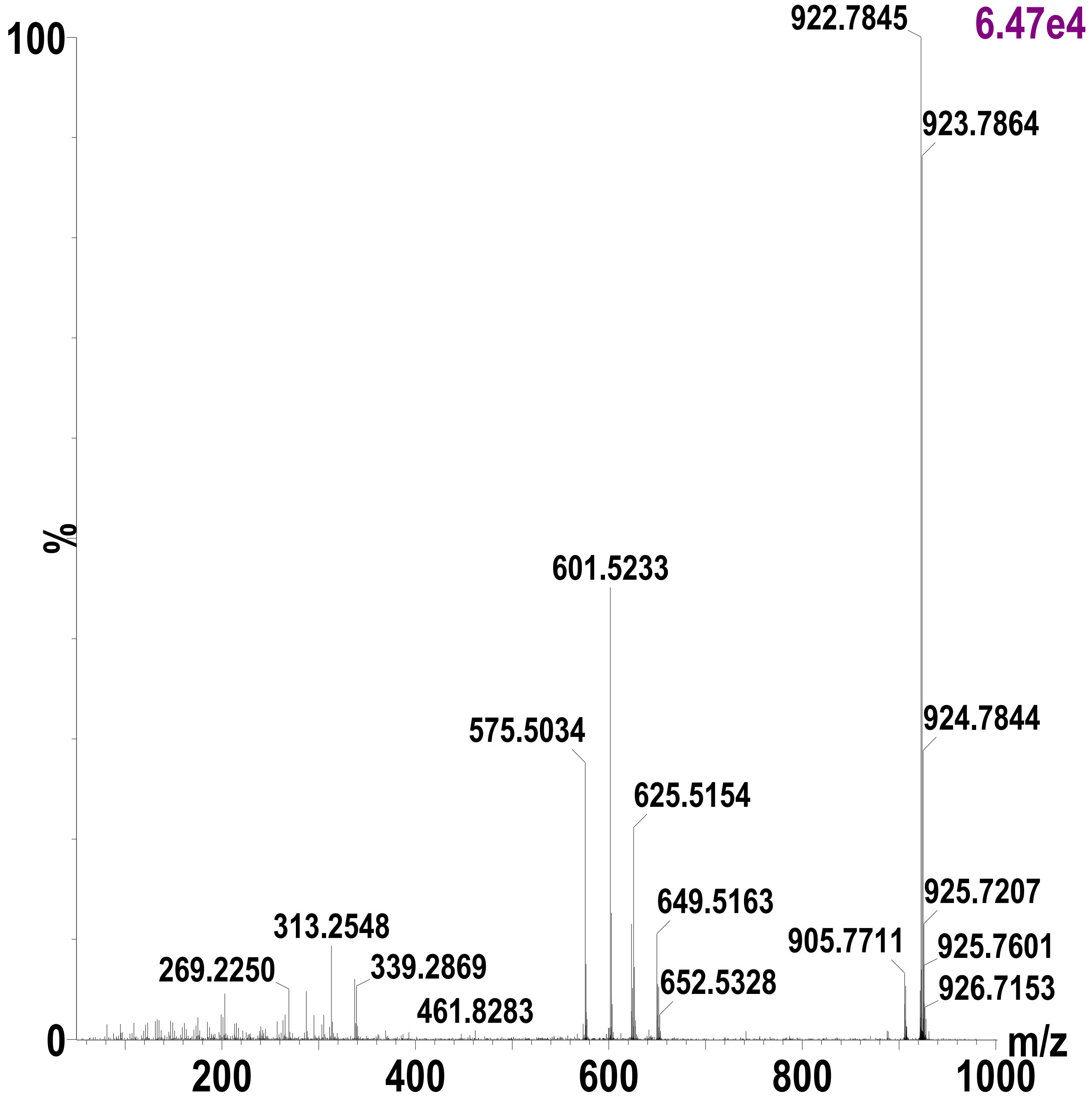

TG (56:8)
Positive Mode
MS/MS

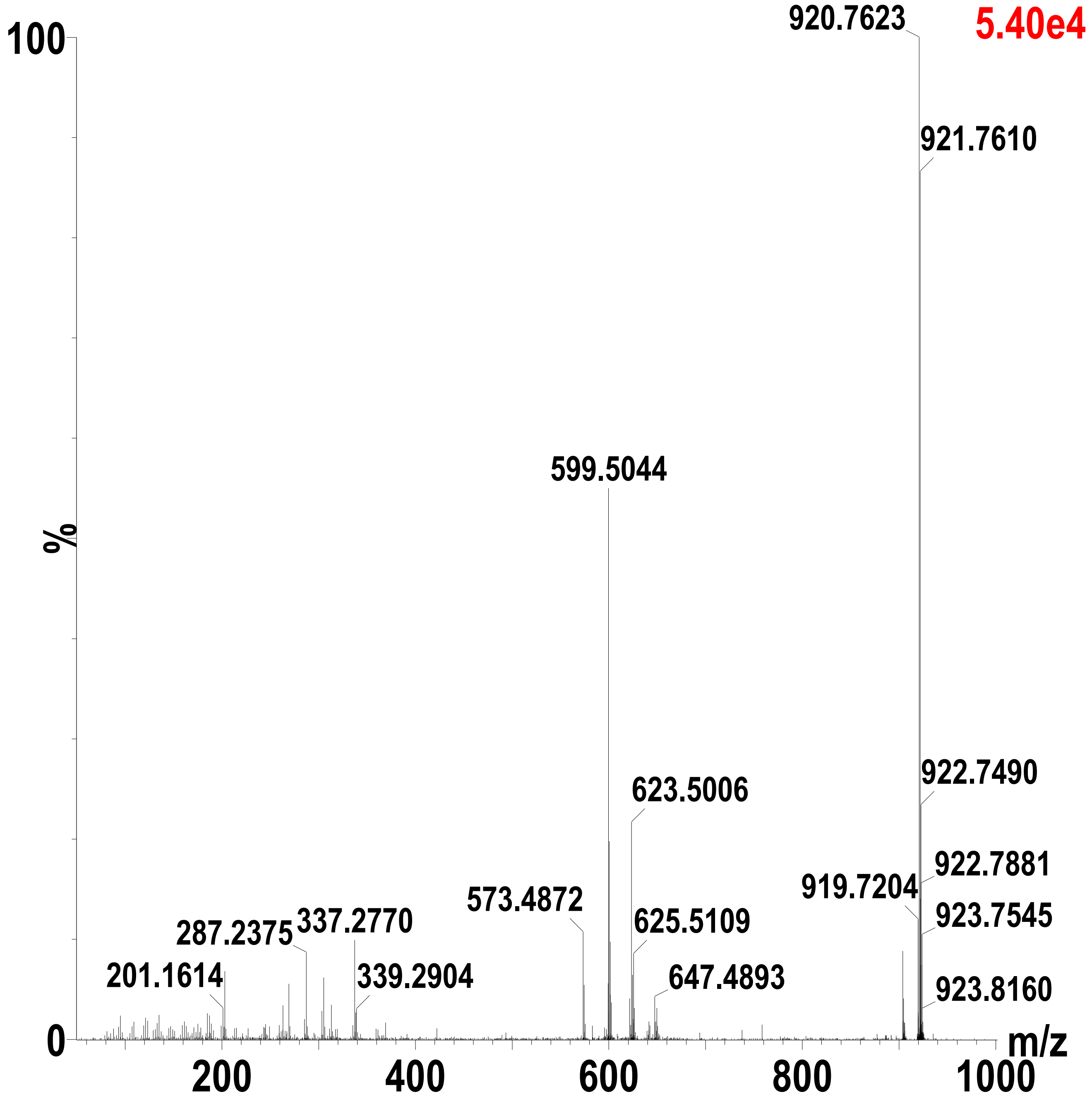

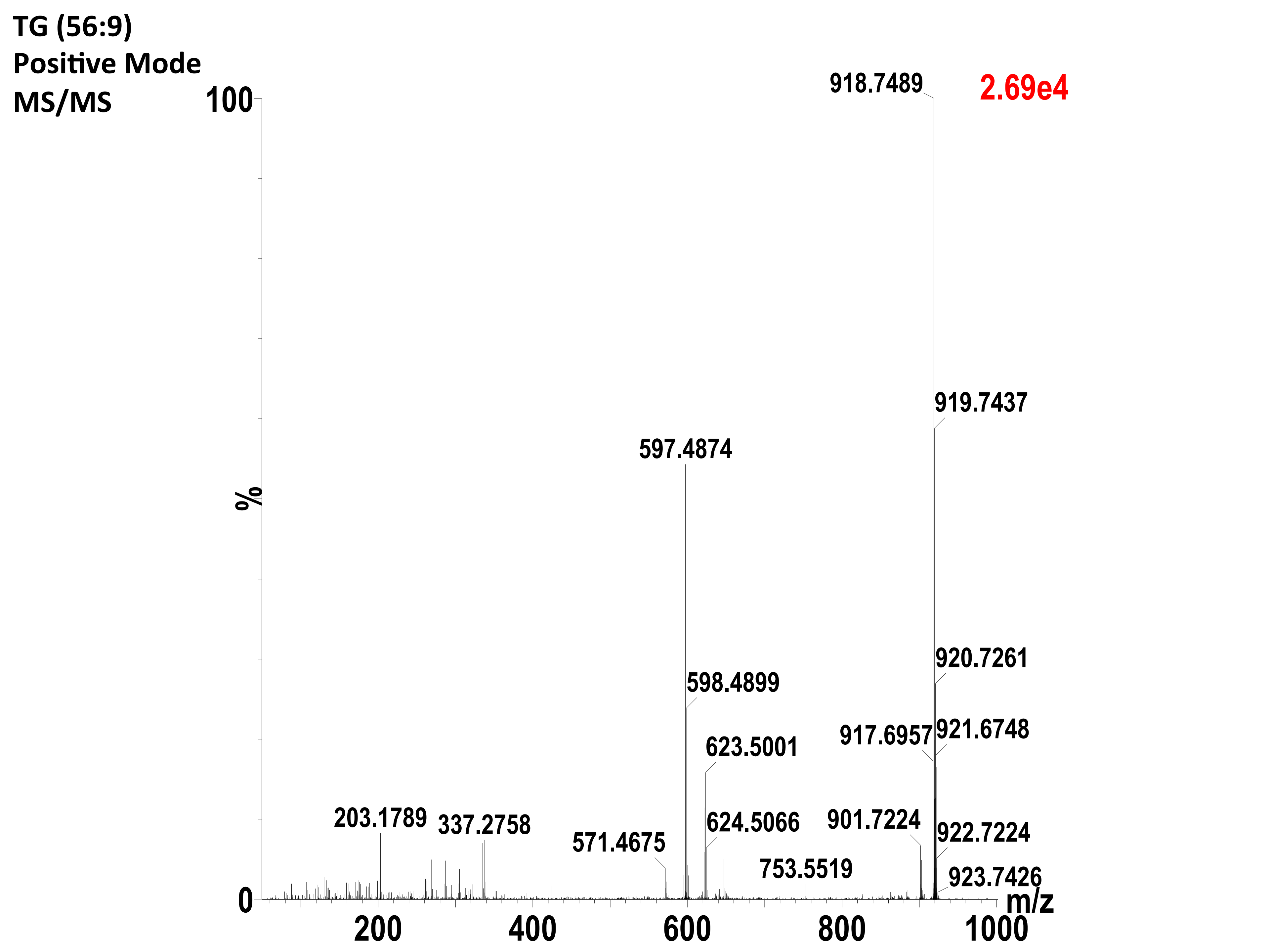
TG (56:9)
Positive Mode
MS/MS
100
%
0
918.7489
2.69e4
919.7437
597.4874
920.7261
598.4899
917.6957
921.6748
623.5001
203.1789
337.2758
571.4675
624.5066
901.7224
922.7224
753.5519
923.7426
200
400
600
800
1000
m/z

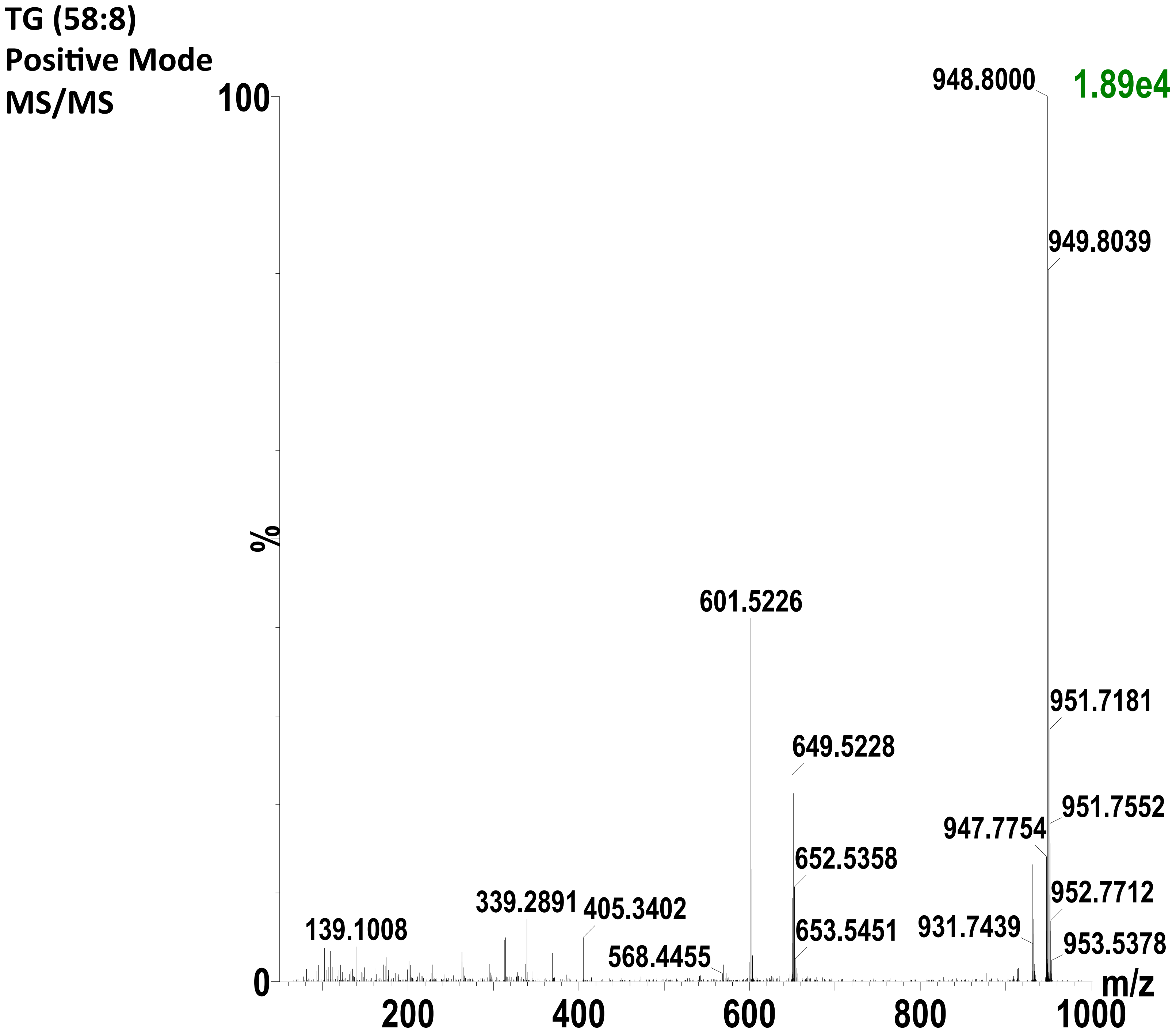
TG (58:8)
Positive Mode
MS/MS
1.89e4
100
%
0
948.8000
949.8039
601.5226
951.7181
649.5228
951.7552
947.7754
652.5358
952.7712
339.2891
405.3402
139.1008
931.7439
653.5451
568.4455
953.5378
200
400
600
800
1000
m/z

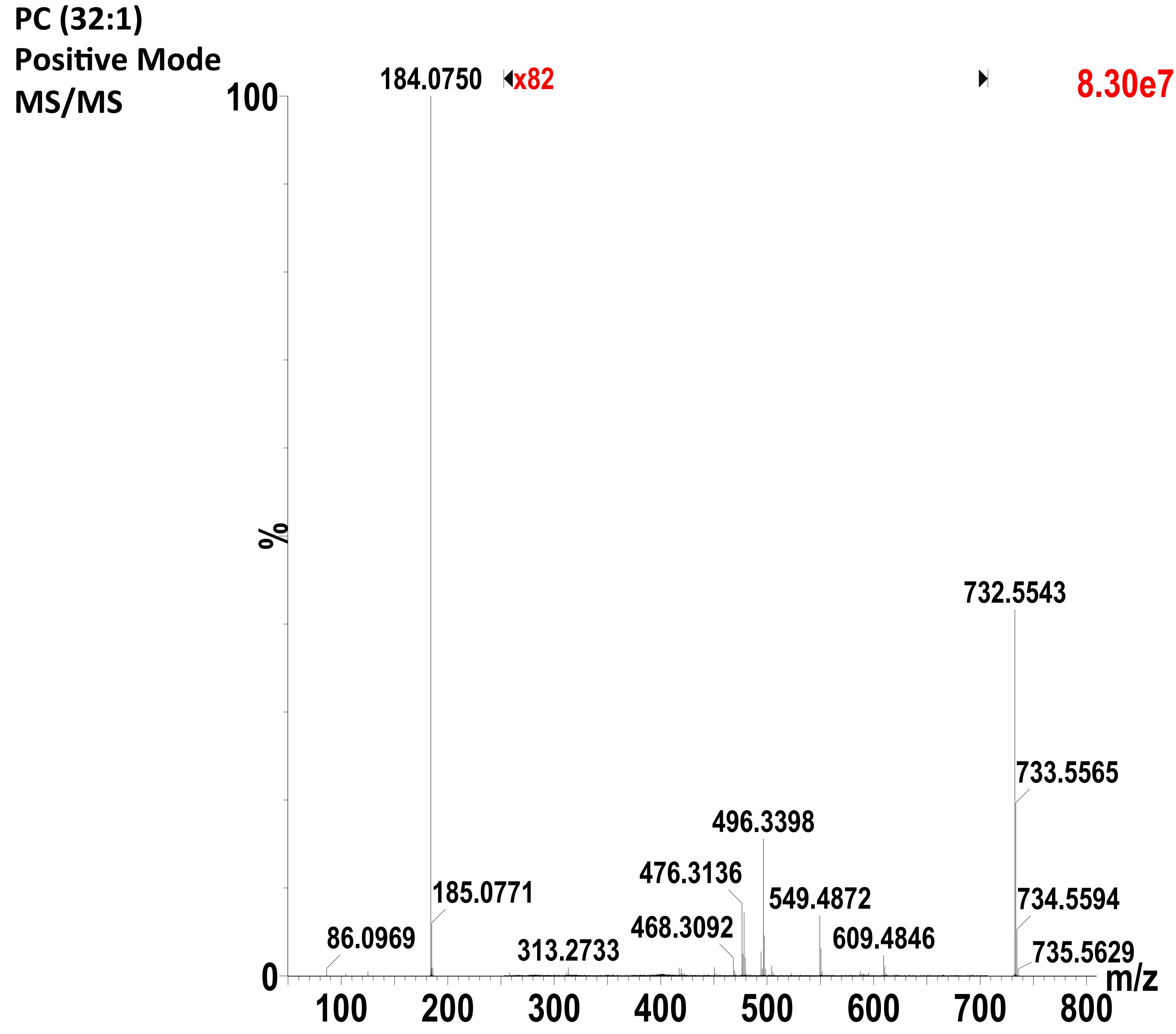
PC (32:1)
Positive Mode
MS/MS
100
184.0750
x82
8.30e7
%
732.5543
733.5565
496.3398
476.3136
185.0771
549.4872
734.5594
468.3092
86.0969
609.4846
313.2733
735.5629
0
m/z
100
200
300
400
500
600
700
800

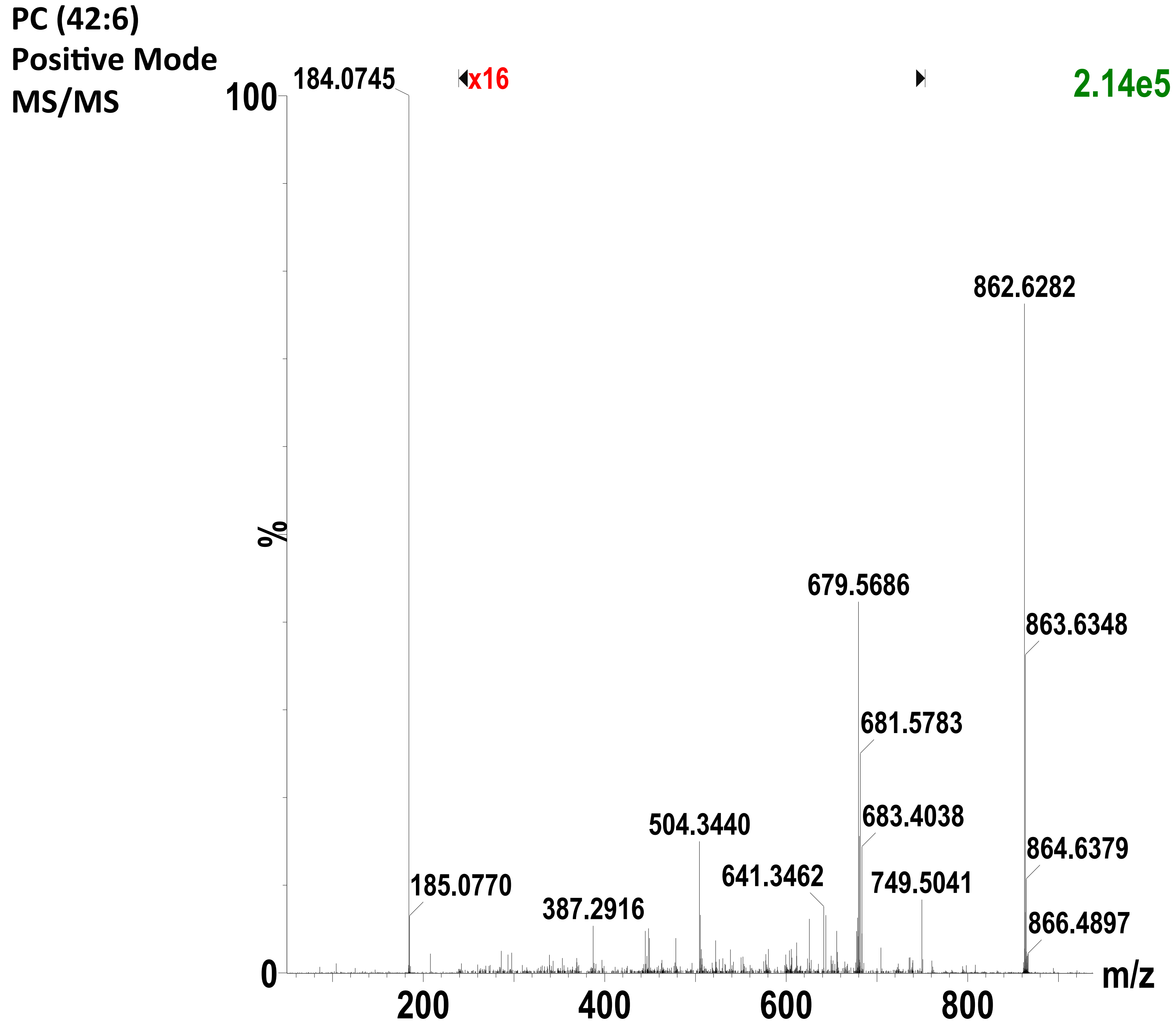

PC (42:6)
Positive Mode
MS/MS
100
0
%
184.0745
x16
2.14e5
862.6282
679.5686
863.6348
681.5783
683.4038
504.3440
641.3462
749.5041
864.6379
185.0770
387.2916
866.4897
200
400
600
800
m/z

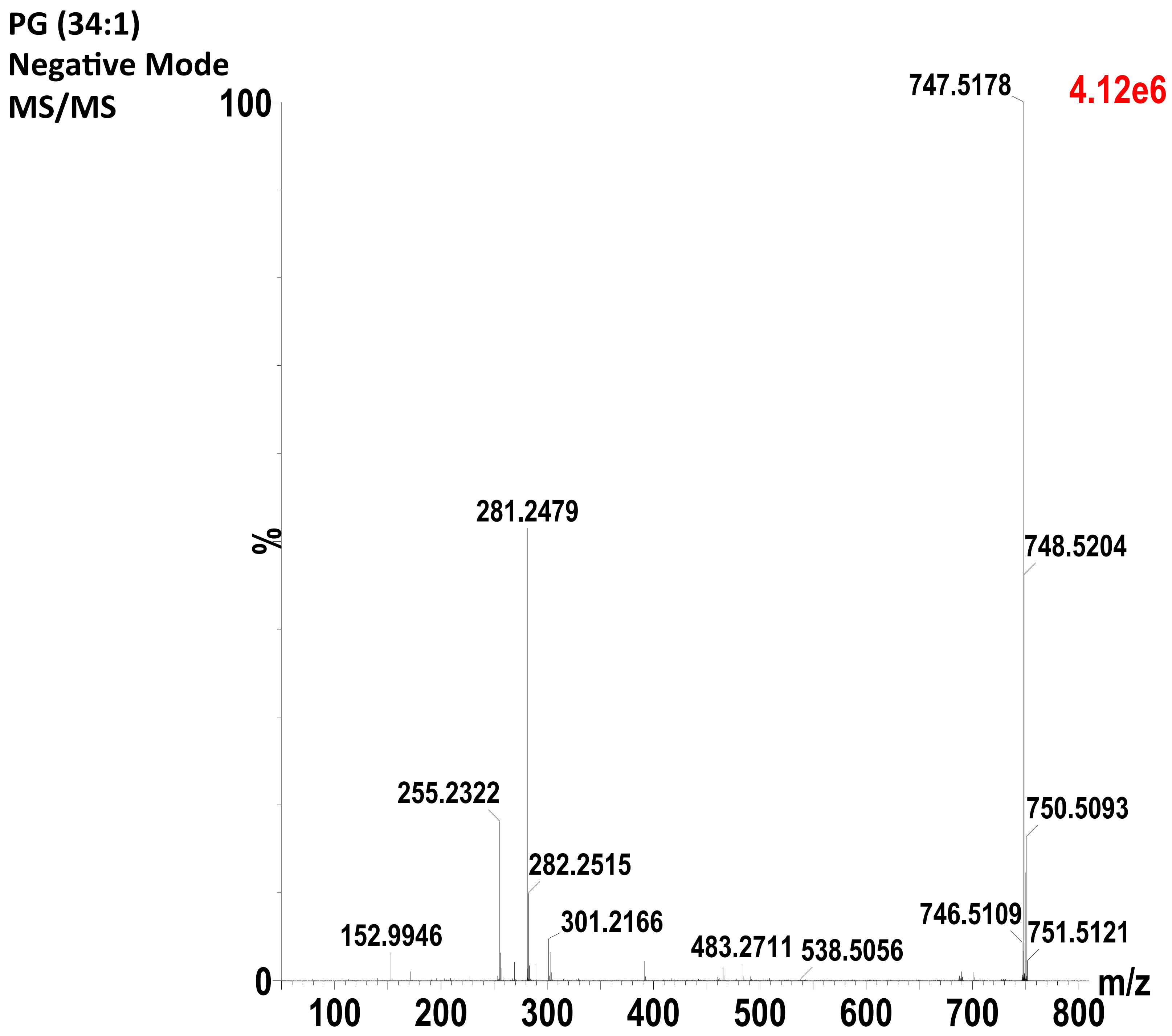
PG (34:1)
Negative Mode
MS/MS
100
0
%
747.5178
4.12e6
281.2479
748.5204
255.2322
750.5093
282.2515
301.2166
746.5109
751.5121
152.9946
483.2711
538.5056
100
200
300
400
500
600
700
800
m/z

Standard
PG (12:0/12:0)
Negative Mode
DIA

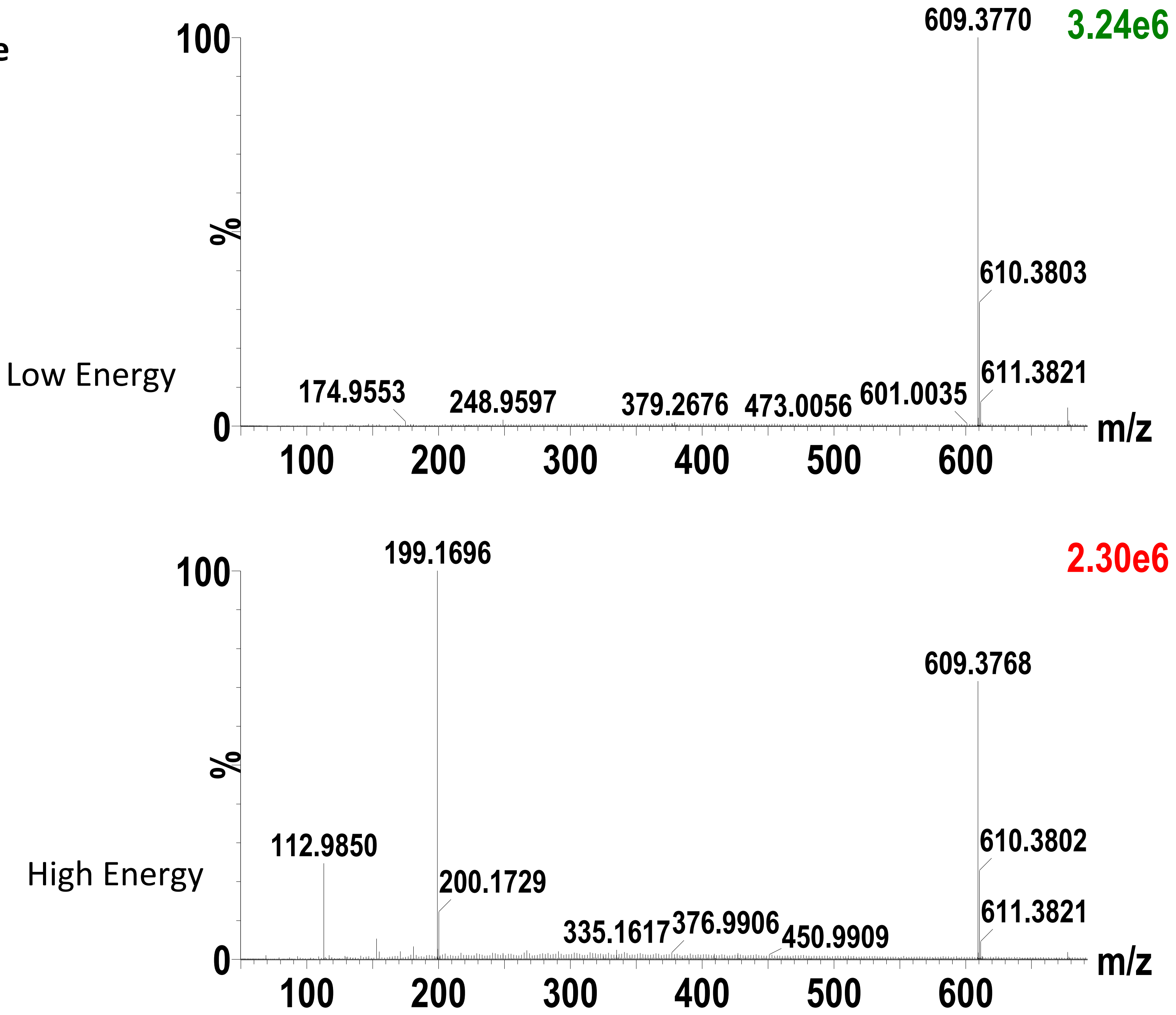

Supplement: Supplementary file 1 — Supplementary Information. [file 41598_2021_90828_MOESM1_ESM.pdf]
